# Herbal medicine for amyotrophic lateral sclerosis: A systematic review and meta-analysis

**DOI:** 10.3389/fphar.2022.946548

**Published:** 2022-08-31

**Authors:** Yuebo Song, Qiuyang Jia, Xiaorui Guan, Sugimoto Kazuo, Jia Liu, Weisong Duan, Luda Feng, Chi Zhang, Ying Gao

**Affiliations:** ^1^ Dongzhimen Hospital, Beijing University of Chinese Medicine, Beijing, China; ^2^ Institute for Brain Disorders, Beijing University of Chinese Medicine, Beijing, China; ^3^ Key Laboratory of Chinese Internal Medicine of Ministry of Education and Beijing, Dongzhimen Hospital, Beijing University of Chinese Medicine, Beijing, China; ^4^ Department of Neurology, The Second Hospital of Hebei Medical University, Shijiazhuang, China; ^5^ Neurological Laboratory of Hebei Province, Shijiazhuang, China

**Keywords:** amyotrophic lateral sclerosis, herbal medicine, motor neuron disease, meta-analysis, alternative and complementary medicine, systematic review

## Abstract

**Background:** The effect of herbal medicine (HM) on amyotrophic lateral sclerosis (ALS) is controversial. Clinical trials investigating HMs continue; however, the use of HM is still questioned. We aimed to systematically review the literature pertaining to the effects and safety of HM in ALS.

**Methods:** Randomised controlled trials (RCTs) that investigated the efficacy of HMs in ALS patients compared to any types of controls were identified. Nine databases and six registers were searched from their inception dates to 25 March 2022. Per the PRISMA guidelines, trials were identified and extracted. The risk of bias was evaluated using the Cochrane’s tool. Certainty of evidence was assessed as per the GRADE criteria. Forest plots were constructed to assess the effect size and corresponding 95% CIs using fixed-effect models, and random-effect models were employed when required. The primary outcome was the activity limitation measured by validated tools, such as the revised ALS Functional Rating Scale.

**Results:** Twenty studies (*N* = 1,218) were eligible. Of these, only five studies were double-blinded, and two were placebo-controlled. Fourteen HMs (fifty-one single botanicals) were involved; *Astragalus mongholicus Bunge, Atractylodes macrocephala Koidz.,* and *Glycyrrhiza glabra L.* were commonly used in nine, eight, and six trials, respectively. For delaying activity limitation, *Jiweiling* injection (MD, 2.84; 95% CI, 1.21 to 4.46; *p* = 0.0006) and *Shenmai* injection (SMD, 1.07; 0.69 to 1.45; *p* < 0.00001) were significantly more efficacious than Riluzole, but the evidence was low quality. For ameliorating motor neuron loss, *Jiweiling* injection [right abductor pollicis brevis (APB): MD, 32.42; 7.91 to 56.93; *p* = 0.01 and left APB: MD, 34.44; 12.85 to 56.03; *p* = 0.002] was favoured, but the evidence was very low quality. Nine studies reported one hundred and twenty-three adverse events, twenty-six of which occurred in the treatment groups and ninety-seven in the control groups.

**Conclusion:** Very low to low quality of evidence suggests that HMs seem to produce superior treatment responses for ALS without increased risk of adverse events. Additional studies with homogeneous participants, reduced methodological issues, and more efficient outcome measures are required to provide confirmatory evidence.

**Systematic Review Registration:** https://www.crd.york.ac.uk/PROSPERO/, identifier CRD42021277443.

## 1 Introduction

As one of the diseases that modifying therapies are urgently needed, the effects of novel treatments such as herbal medicine (HM) on amyotrophic lateral sclerosis (ALS) are being broadly investigated. However, the various responses to HMs were questioned continually. ALS is a fatal, neurodegenerative disorder characterized by the progression of focal muscle weakness and wasting until respiratory failure within 3–5 years (Brown and Al-Chalabi, 2017). Motor neuron loss triggers activity limitation and reduces the quality of life (QOL) of patients with ALS. Currently, only two disease-modifying drugs are approved by the Food and Drug Administration for ALS treatment. Riluzole only slightly prolongs survival (Miller et al., 2002), and Edaravone is efficacious merely in patients who meet strict eligibility criteria (Abe et al., 2017). Because of the modest benefits of current therapies, many patients with ALS resort to HMs.

According to a cross-sectional survey in China, the proportion of herbal users among patients with ALS exceeds 90% (Pan et al., 2013b), and the corresponding proportion is 40% in America (Vardeny and Bromberg, 2005). Nevertheless, the HMs get both praise and blame along with their widespread use. The effects of HMs have been continuously praised in ALS animal models and *in vitro*. *Bojungikgi* formula improved muscle and spinal cord function (Cai et al., 2019), *Shenqi Fuzheng* injection extended the overall survival and improved the pathological manifestations in the brain (Sugimoto et al., 2021), and *Huoling Shengji* formula significantly prolonged lifespan and prevented motor neuron loss (Zhou et al., 2018). Additionally, published clinical trials have enhanced the credibility of the experimental evidence. However, many RCTs of proposed HMs have failed to show positive results in the past 20 years. Furthermore, certain HMs have been linked with poorer prognosis in patients with ALS in a single-centre cohort study (Chen et al., 2015). This phenomenon confuses both the researchers and patients about whether the effect of HM is fair or whether it should be blamed for the design of RCTs for ALS.

Consequently, we performed a systematic review and meta-analysis of the published literature to explore whether HM may usefully improve the activity limitation and whether the safety evidence of HM for ALS can be established.

## 2 Methods

### 2.1 Protocol and registration

This systematic review was registered prospectively with PROSPERO: CRD42021277443. We followed the Preferred Reporting Items for Systematic Reviews and Meta-Analysis (PRISMA) guidelines (Page et al., 2021). Ethical approval was not required for this study.

### 2.2 Eligibility criteria

#### 2.2.1 Types of studies

The RCTs were included irrespective of language and publication status.

#### 2.2.2 Types of participants

Adults diagnosed with ALS, regardless of sex or ethnicity, were eligible. The diagnostic criteria based on all versions of consensus criteria including the El Escorial criteria (Brooks, 1994), the revised El Escorial criteria (Brooks et al., 2000), the Awaji algorithm (de Carvalho et al., 2008), or the Gold Coast criteria (Shefner et al., 2020) were acceptable. The adapted diagnostic criteria on the basis of these standards and commonly used in various countries were allowed.

#### 2.2.3 Types of interventions

HMs in any form were included. Our definition of HMs includes herbs, herbal materials, herbal preparations, and finished herbal products that contain active ingredient parts of plants, other plant materials, or combinations, according to the World Health Organization (The World Health Organization, 2021). The comparators could be as follows: placebo, other pharmacological interventions such as Riluzole or Edaravone, or non-pharmacological intervention such as acupuncture or massage, when these interventions were administered as comparators or equally to all arms in trials.

#### 2.2.4 Types of outcome measures

Any effect-related outcomes were measured. The primary outcome was activity limitation, measured with validated instruments such as the ALS Functional Rating Scale (ALSFRS) (The ALS CNTF treatment study (ACTS) phase I-II Study Group, 1996), revised ALSFRS (ALSFRS-R) (Cedarbaum et al., 1999), or the modified Norris Scale (Norris et al., 1974). The secondary outcomes included tracheostomy-free survival (Paganoni et al., 2014) or overall survival, loss of strength (respiratory muscles and limb muscles), QOL, functional status, motor neuron loss, measurements based on traditional medicine theory, and pharmacodynamic biomarkers. The tracheostomy-free survival is defined as time to death, tracheostomy, or permanent non-invasive positive pressure ventilation, which shows end-of-life care for patients with ALS (Paganoni et al., 2014). The deficits of respiratory muscles are commonly assessed *via* forced vital capacity (FVC), and limb muscles are quantitatively evaluated by hand-held dynamometry (HHD) or Medical Research Council Scale (MRC). The change of QOL captured using validated instruments, such as the ALS Assessment Questionnaire-40 (ALSAQ-40) (Jenkinson et al., 1999), the ALS Specific Quality of Life-revised (ALSSQOL-R) (Simmons et al., 2006), the MOS Item Short-form Health Survey (SF-36) (Ware and Sherbourne, 1992), or the Barthel index, during the treatment was evaluated. Validated scales assessing the functional status, such as the Appel ALS Score (AALSS) (Appel et al., 1987), were included. The motor neuron loss measured via motor unit number estimation (MUNE) or other neurophysiological tests was assessed. The MUNE is a measure of remaining motor units and, therefore, an indirect measure of motor neuron loss. Any data of pharmacodynamic biomarkers such as the neurofilament light chain were abstracted. For safety assessment, any adverse events (AEs) and serious adverse events (SAEs) were summarized.

### 2.3 Search strategy

Nine databases, including MEDLINE, Embase, Cochrane Central Register of Controlled Trials (CENTRAL), Web of Science, China National Knowledge Infrastructure (CNKI), Wanfang Data, SinoMed, National Institute of Informatics Support Academic Information Services (CiNii), and Korean Journal Database (KCL), were retrieved respectively from their inception dates to 25 March 2022. Relevant grey literature sources such as reports, dissertations, theses, and conference abstracts were identified to reduce the risk of publication bias.

The on-going trials and unpublished studies were searched *via* the following registers: ClinicalTrials.gov; the World Health Organization International Clinical Trials Registry Platform (ICTRP); Chinese Clinical Trial Registry (ChiCTR); EU Clinical Trials Register; Clinical Research Information Service (CRiS), Republic of Korea; Japan Primary Registries Network (JPRN).

The searched terms were as follow: “amyotrophic lateral sclerosis,” “motor neuron disease,” “Lou Gehrig’s disease,” “Charcot disease,” “phytotherapy,” “traditional medicine,” “medicinal plant,” “herbal medicine,” “plant extract,” “plant preparation,” “traditional Chinese medicine,” “Chinese drug,” “Chinese formul*,” “Chinese prescri*,” “kampo medicine,” “Chinese materia medica,” “japanese medicine,” “japanese drug,” “japanese formul*,” “japanese prescri*,” “korean medicine,” “korean drug,” “korean formul*,” “korean prescri*,” and “randomised controlled trial.” The search strategies are listed in Supplementary Appendix A1. To highly identify RCTs, the Cochrane sensitivity-maximizing filter for RCTs (2008 revision in Ovid format) was adopted (Lefebvre et al., 2021).

### 2.4 Study selection and data extraction

According to prespecified selection criteria, two authors (YBS and XRG) reviewed the titles and abstracts of retrieved articles after duplicates were removed. The articles that did not fulfil the inclusion criteria were removed. The remaining articles were screened with full text by the same two authors independently. Any disagreements in primary and full-text screening were discussed to be resolved. A third review author (CZ) was consulted if required. All exclusion reasons were recorded.

For eligible articles remaining after the primary and full-text screening, two authors (YBS and QYJ) extracted the eligibility criteria, study design, participants, interventions, comparators, outcomes, results, and other relevant information using standard data extraction templates. For studies reporting results at more than one time point, the final data of the intended treatment period was mainly extracted. The same scheme resolved the disagreements. The multiple publications of the same study were listed under the original article. The missing information from the included studies was obtained via contact with the authors to reduce the reporting biases. The reference lists of all relevant primary studies were checked for other potential studies.

In addition to identifying potential benefits, possible AEs were also extracted, including liver injury, kidney damage, gastrointestinal dysfunction, allergy, skin discomfort, cardiovascular events, and any SAEs.

### 2.6 Strategy for data synthesis

Statistical analysis was performed using software provided by the Cochrane Collaboration (Review Manager 5.3). Relevant characteristics of studies were compared to assess which studies were eligible for each synthesis (Table 1). For continuous outcomes, the mean difference (MD) or standardized mean difference (SMD) with 95% confidence interval (CI) was calculated depending on the similarity of outcome measurements. MD would be selected when studies all reported the outcome using the same scale. The relative risk (RR) with a 95% CI was calculated for dichotomous outcomes. Trials were excluded from the synthesis when essential data were missing.

**TABLE 1:** Characteristics of included studies.

Study	Certainty of ALS^a^	Participant		Tested treatment (TT)	Allocation		Duration, months	Outcome measures	Study design
		Treated arm N; Age (SD), years	Control arm N; Age (SD), years		Treated arm	Control arm			
Bao et al. (2016)	A + B + C + D	24; unclear	24; unclear	*Jiawei Sijunzi* formula	TT + conventional treatment	conventional treatment	6	STMS, AE	OL
Cai (2011)	Unclear	20; 51.2 (11.7)	18; 48.5 (9.7)	*Shenmai* injection	TT + conventional treatment	conventional treatment	1.8^b^	ALSFRS/ALSFRS-R^c^	OL
Chico et al. (2018)	A + B + C + D	18; 58.2^d^	24; 65.6^d^	Curcumin	TT	placebo	3	ALSFRS-R, HHD, MRC, PB, AE	DB
Fang (2016)	A + B + D	30; 49.1 (11.3)	30; 53.4 (9.7)	*Jianpi Yifei* formula	TT	Riluzole	3	ALSFRS-R, ALSAQ-40 (sub), AE	OL
Jin (2013)	A + B + D	15; 57.8 (10.6)	13; 50.5 (18.9)	*Guilu Erxian* glues + acupuncture	TT + Riluzole	Riluzole	6	ALSFRS-R, STMS, AE	OL
Li et al. (2011)	A + B + C + D	30; 56.4 (11.1)	28; 55.8 (10.7)	*Fuyuan Shengji* granule	TT + Riluzole	Riluzole	6	Modified Norris Scale, FVC, tracheostomy-free survival	OL
Li (2019a)	A + B + D	39; 52.4 (11.5)	39; 51.1 (10.3)	*Jianpi Yifei* formula + massage	TT + conventional treatment	conventional treatment	3	ALSFRS, AALSS, STMS	OL
Ma (2006)	A + B + D + E	30; 48.3 (10.2)	30; 48.7 (11.1)	*Jiweiling* injection	TT + dummy Riluzole	Riluzole + dummy TT	3	ALSFRS, Modified Norris Scale, AALSS, ALSAQ-40, FVC, VC, MUNE, AE	DB
Pan et al. (2013a)	A + B	24; 51.6 (7.2)	24; 50.1 (4.2)	*Jiawei Sijunzi* formula	TT	Riluzole	6	ALSFRS-R, MRC, SF-36 (sub), AE	OL
Pan (2015)	A + B + D	40; 49.4 (9.0)	40; 50.1 (8.1)	*Shenzhe Jiangqi* powder	TT + dummy Riluzole	Riluzole + dummy TT	3	ALSFRS, Modified Norris Scale, ALSAQ-40, MUNE, AE	DB
Riva et al. (2019)	A + B + C	30; 58.4 (10.6)	30; 57.2 (13.8)	Cannabinoids	TT	placebo	1.5	ALSFRS-R, FVC, MRC, Barthel index, AE	DB
Su et al. (2006)	A + B + C + D	25; 60.2 (14.1)	10; 59.4 (9.0)	*Yiqi Qiangji* formula	TT + Riluzole	Riluzole	3	Modified Norris Scale, STMS, CMAP	OL
Sui et al. (2016)	A + B + C + D	33; 54 (12.0)	31; 54 (11.9)	*Huoling Shengji* formula	TT	Riluzole	3	Modified Norris Scale, STMS, AE	OL
Wang (2007)	A + B + C + D	30; 44.6 (9.6)	30; 48.1 (8.5)	*Jiweiling* injection	TT	Riluzole	3	ALSFRS, ALSAQ-40, FVC, VC, MUNE, PB, AE	OL
Wang et al. (2009)	A + B + C + D	100; 55.1 (13.5)	25; 56.6 (11.2)	*Fuyuan Shengji* granule	TT	Riluzole	3	Modified Norris Scale, STMS, CMAP, PB, AE	OL
Wang 2017a	A + B + C + D	30; unclear	30; unclear	*Jianpi Yifei* formula	TT + Riluzole	Riluzole	2	ALSFRS-R, MRC, FVC, AE	OL
Wang 2017b	A + B + C + D	30; 46.2 (7.0)	30; 48.8 (3.8)	*Zishen Jianpi* formula	TT + conventional treatment	conventional treatment	1	ALSFRS, Barthel index, STMS, AE	OL
Xv et al. (2011)	A + B + D	40; 60^e^	40; 57^e^	*Bushen Jianpi Shugan* formula	TT	Riluzole	6	Modified Norris Scale, STMS	OL
Zhang (2020)	A + B + D	42; 45.4 (6.5)	42; 45.5 (6.4)	*Shenmai* injection	TT + conventional treatment	conventional treatment	1.8^b^	ALSFRS-R	OL
Zhu (2016)	A + B + C + D	25; unclear	25; unclear	*Jiawei Sijunzi* formula	TT	1/10 dose of TT	9	ALSFRS-R, STMS, AE	DB

AALSS, appel amyotrophic lateral sclerosis score; AE, adverse event; ALSAQ-40, amyotrophic lateral sclerosis assessment questionnaire-40; ALSFRS, amyotrophic lateral sclerosis functional rating scale; ALSFRS-R, amyotrophic lateral sclerosis functional rating scale-revised; CMAP, compound muscle action potential; DB, double-blind; FVC, forced vital capacity; HHD, hand-held dynamometry; MRC, medical research council scale; MUNE, motor unit number estimation; OL, open-label; PB, pharmacodynamical biomarker; SD, standard deviation; SF-36, MOS item short-form health survey-36; STMS, score of traditional medicine syndrome; TT, tested treatment; VC, vital capacity.

a Certainty of ALS: A. definite, B. probable, C. laboratory-supported probable, D. possible, E. suspected.

b (treatment for 15 days + wash out for 3 days)×3 cycles.

c The version of amyotrophic lateral sclerosis functional rating scale is unclear.

d Did not reported the SD.

e Reported in median.

Both random-effect models and fixed-effect models were performed in meta-analysis when available. The results from both models were reported when significant heterogeneity existed, and the heterogeneity was tried to explain by subgroup if applicable. When there was no significant heterogeneity, the results of the fixed-effect model were reported. When the heterogeneity was substantial, both models were abandoned, and the meta-analysis was replaced by the qualitative summary. The heterogeneity was calculated with the I^2^ test. To visually display the results of syntheses, forest plots were constructed. We planned the subgroups classified by disease course and different durations of intervention. We projected to perform the sensitivity analysis using the following filters: certainty of ALS and risk of bias.

### 2.5 Risk of bias assessment

Two review authors (YBS and QYJ) assessed the risk of bias independently using the Cochrane Handbook for Systematic Reviews of Interventions (Revised Cochrane risk-of-bias tool for randomised trials) for eligible studies (Sterne et al., 2019). The bias domain of the randomization process, deviations from intended interventions, missing outcome data, measurement of the outcome, selection of the reported result, and other biases were evaluated. A judgment of bias was made and divided into “low risk,” “high risk,” and “some concerns.” Unclear items in studies were further checked by contacting the corresponding authors. Any disagreements were discussed with a third reviewer (CZ). Funnel plots were constructed to evaluate the publication bias across studies when at least ten studies were included in the quantitative analysis synthesis.

The Grading of Recommendations Assessment, Development, and Evaluation (GRADE) system was used by two reviewers (QYJ and YBS) to separately assess the certainty of evidence for each outcome. The discrepancies were resolved by discussion with a third reviewer (XRG).

## 3 Results

### 3.1 Results of the search

**FIGURE 1:**
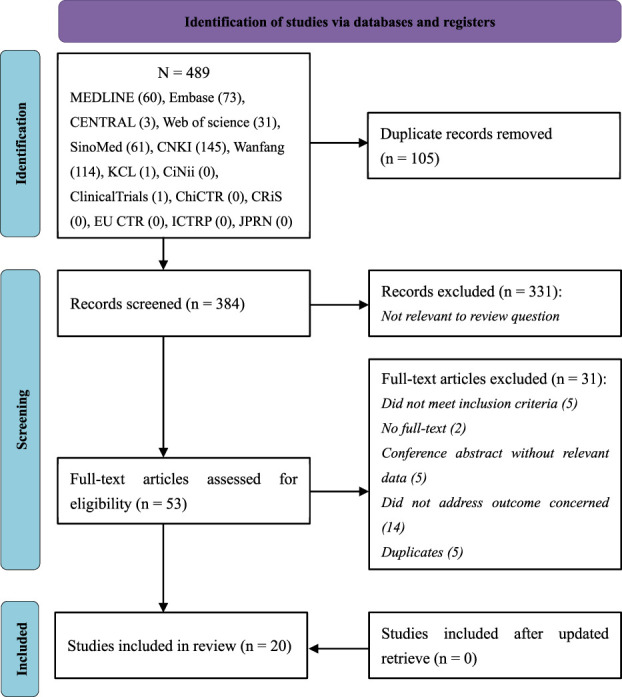
PRISMA flow diagram showing literature search results. *PRISMA*, Preferred reporting items for systematic reviews and meta-analysis.

A total of 489 records were retrieved. Of these trials, one was identified from the register and has been completed. 384 records remained after duplicates were removed. After titles and abstracts were screened, 331 records were excluded due to non-clinical trials, non-herbal interventions, or non-ALS participants. After reading the full text, 31 trials were excluded, and the reasons were listed in Supplementary Appendix A2. Before the manuscript was submitted, we updated retrieval, but no additional trials meeting inclusion criteria were found. Therefore, 20 RCTs were included. The PRISMA flowchart of study selection is presented in Figure 1.

### 3.2 Included studies

The characteristics of 20 included RCTs are listed in Table 1. All included trials adopted parallel two-arm designs. Five studies were double-blinded (Ma, 2006; Pan, 2015; Zhu, 2016; Chico et al., 2018; Riva et al., 2019), and other studies were open-labelled. Three trials were published in English (Pan et al., 2013a; Chico et al., 2018; Riva et al., 2019), and others were reported in Chinese. Two included studies were conducted in Italy (Chico et al., 2018; Riva et al., 2019), and the rest were in China. Only three trials reported the sample size calculation (Fang, 2016; Zhu, 2016; Riva et al., 2019).

### 3.3 Participants

The number of participants in each included trial ranged from 28 to 125, with a total of 1,218 investigated subjects (655 were in the treatment groups, and 563 were in the control groups). Of them, 838 (68.8%) participants were male sex. There were nine trials including subjects on the basis of both ALS diagnostic criteria and traditional medicine signs. Two trials did not describe inclusion and exclusion criteria (Su et al., 2006; Cai, 2011). One trial did not report whether baseline characteristics were well matched between groups or not (Su et al., 2006).

### 3.4 Interventions

Among the twenty included trials, fourteen HMs (Supplementary Appendix A3) were investigated. Most were herbal preparations, and only four were patent herbal productions (Cannabinoids, Curcumin, *Jiweiling* injection, and *Shenmai* injection). One HM was delivered *via* an oromucosal spray (Riva et al., 2019), two were administered intravenously (Ma, 2006; Wang, 2007; Cai, 2011; Zhang, 2020), and the other eleven HMs were taken orally. The fourteen HMs contained fifty-one single botanicals (Supplementary Appendix A3); *Astragalus mongholicus Bunge, Atractylodes macrocephala Koidz., Glycyrrhiza glabra L., Poria cocos (Schw.) Wolf.,* and *Codonopsis pilosula (Franch.) Nannf.* Were commonly used in nine, eight, six, six and five trials, respectively. One trial did not describe the procedure of medicine preparation. One trial investigated HM combined with acupuncture (Jin, 2013), and another combined with massage (Li P., 2019). The duration of treatments ranged from 1 month to 9 months, and most trials (nine trials) investigated participants for 3 months (Table 1).

### 3.5 Comparators

Two studies compared HM with placebo (Chico et al., 2018; Riva et al., 2019). Eight studies compared HM with Riluzole (Ma, 2006; Wang, 2007; Wang et al., 2009; Xv et al., 2011; Pan et al., 2013a; Pan, 2015; Fang, 2016; Sui et al., 2016). Four studies conducted Riluzole add-on therapy for ALS (Su et al., 2006; Li et al., 2011; Jin, 2013; Wang et al., 2017). Five studies adopted conventional treatment add-on therapy (Cai, 2011; Bao et al., 2016; Wang, 2017; Li, 2019b; Zhang, 2020). One study used 1/10 dose of investigated medicine as a placebo, thus was classified as a dose-response controlled trial (Zhu, 2016) (Table 1).

### 3.6 Risk-of-bias assessment

**FIGURE 2:**
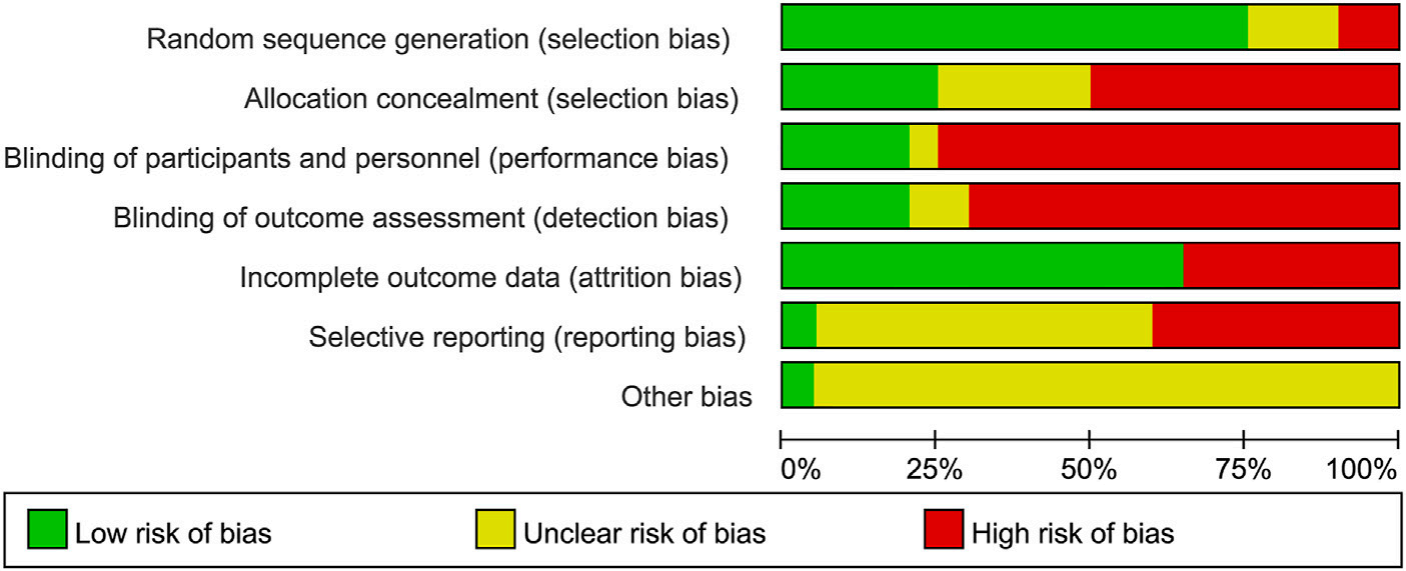
Judgments about each risk of bias item presented as percentages across all included studies.

**FIGURE 3:**
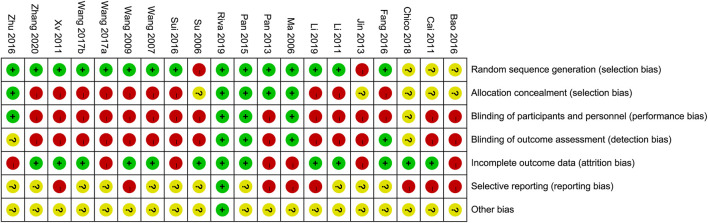
Judgments about each risk of bias item for each included study.

The quality assessment of each study’s random sequence generation, allocation concealment, blinding, incomplete outcome data, selective reporting, and other bias are presented in Figures 2, 3. We were not able to detect publication bias for any analysis.

### 3.7 Effects

#### 3.7.1 Activity limitation

A total of 19 studies (*N* = 1,045, Supplementary Appendix A4) measured activity limitation. Significant differences were found between intervention and controls.

Fourteen trials reported changes in activity limitation measured with the ALSFRS-R or the ALSFRS. As the modified Norris Scale is less sensitive than the ALSFRS-R, it has fallen out of favour (Paganoni et al., 2014). But five trials still employed it as the measure of activity limitation (Su et al., 2006; Wang et al., 2009; Li et al., 2011; Xv et al., 2011; Sui et al., 2016). Two trials used both the ALSFRS and modified Norris Scale (Ma, 2006; Pan, 2015).

Of the 19 trials, nine reported better effects of treatment than controls (Supplementary Appendix A4), among which four trials employed the ALSFRS-R (Jin, 2013; Zhu, 2016; Wang et al., 2017; Zhang, 2020); three trials measured with the ALSFRS (Ma, 2006; Wang, 2007; Pan, 2015), even though these trials were conducted after the ALSFRS-R (revised version of ALSFRS) has been applied in clinical studies regularly; one trial did not mention the accurate version of ALS Function Rating Scale (Cai, 2011); one trial measured with the modified Norris Scale (Li et al., 2011).

**FIGURE 4:**
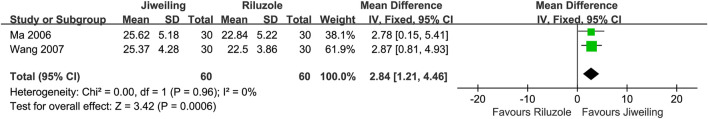
Effect of *Jiweiling* injection on activity limitation in patients with amyotrophic lateral sclerosis.

**FIGURE 5:**
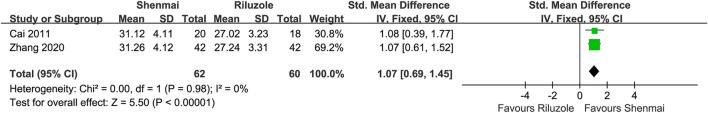
Effect of *Shenmai* injection on activity limitation in patients with amyotrophic lateral sclerosis.

In pooling analysis, fixed-effect models were used and were good fits to the data. For delaying activity limitation, *Jiweiling* injection ([Araliaceae*; Panax ginseng C. A. Mey.*] and [Apiaceae*; Angelica sinensis (Oliv.) Diels*]) (MD, 2.84; 95% CI, 1.21 to 4.46; *p* = 0.0006) (Ma, 2006; Wang, 2007) and *Shenmai* injection ([Araliaceae*; Panax ginseng C. A. Mey.*] and [Asparagaceae*; Ophiopogon japonicus (Thunb.) Ker Gawl.*]) (SMD, 1.07; 0.69 to 1.45; *p* < 0.00001) (Cai, 2011; Zhang, 2020) were significantly more efficacious than controls (Figures 4, 5). However, the insufficient number of homogeneous trials did not allow the subgroup analysis and sensitivity analysis.

Another 10 trials showed no statistically significant differences between treatments and controls. Four of them were measured with the ALSFRS-R (Pan et al., 2013a; Fang, 2016; Chico et al., 2018; Riva et al., 2019) and 2 with ALSFRS (Wang, 2017; Li P., 2019). In the Curcumin trial (Chico et al., 2018), the author also analysed the sub scale of the ALSFRS-R (question 10–12) for respiratory assessment, and the significant result was found. However, this article did not report any specific data for synthesis. Another three trials measured the modified Norris Scale (Su et al., 2006; Wang et al., 2009; Xv et al., 2011; Sui et al., 2016).

#### 3.7.2 Survival

Only one trial (*N* = 58, follow-up for 18 months, Supplementary Appendix A4) reported tracheostomy-free survival but did not show a significant difference between treatment and control (Jin, 2013). This trial displayed the Kaplan-Meier survival curve but did not report the Hazard ratio and 95% CI.

#### 3.7.3 Loss of strength

Seven studies (*N* = 738, Supplementary Appendix A4) assessed the loss of strength. Meta-analysis was replaced by the qualitative summary due to substantial heterogeneity [I^2^ = 75% in pooling analysis of respiratory function measured with FVC; I^2^ = 81% when measured with the vital capacity (VC)].

Five trials measured FVC (Ma, 2006; Wang, 2007; Li et al., 2011; Wang et al., 2017; Riva et al., 2019). Two of them showed statistical significance in delaying the decline of ventilatory muscle strength (Ma, 2006; Wang, 2007). These two trials also showed significant differences when measuring VC. Another three trials showed no statistical significance (Li et al., 2011; Wang et al., 2017; Riva et al., 2019).

Four trials evaluated MRC (Pan et al., 2013a; Wang et al., 2017; Chico et al., 2018; Riva et al., 2019), and all of them did not show significant effects. Of these, the Curcumin trial (Chico et al., 2018) also used HHD to measure the accurate grip force.

#### 3.7.4 Quality of life

Seven trials (*N* = 394, Supplementary Appendix A4) evaluated the QOL. Three reported significant difference between treatment and control when measuring the ALSAQ-40 (Ma, 2006; Wang, 2007; Pan, 2015). Additionally, one trial measuring the subscale of ALSAQ-40 reported no significant effect (Fang, 2016).

Two trials employed the Barthel index; one showed a significant effect (Wang, 2017), and another showed no significant difference (Riva et al., 2019). One trial (Pan et al., 2013a) reported results on the subscale of the SF-36 and found no significant improvement.

#### 3.7.5 Functional status

Two trials (*N* = 138, Supplementary Appendix A4) assessed the functional status of ALS patients *via* the AALSS. Of them, one showed a statistically significant difference (Ma, 2006), and another reported no significant effect (Li, 2019b).

#### 3.7.6 Score of traditional medicine syndrome

Nine trials (*N* = 560, Supplementary Appendix A4) recorded the change in symptoms and signs based on traditional medicine theory (Su et al., 2006; Wang et al., 2009; Xv et al., 2011; Jin, 2013; Bao et al., 2016; Sui et al., 2016; Zhu, 2016; Wang, 2017; Li, 2019b), and only one of them reported no significant improvement compared to control (Li, 2019b). However, most measurements used in these trials lack verification of reliability and validity.

#### 3.7.7 Motor neuron loss

Three trials (*N* = 170, Supplementary Appendix A4) reported the change in MUNE (Ma, 2006; Wang, 2007; Pan, 2015) and all showed statistical significance.

**FIGURE 6:**
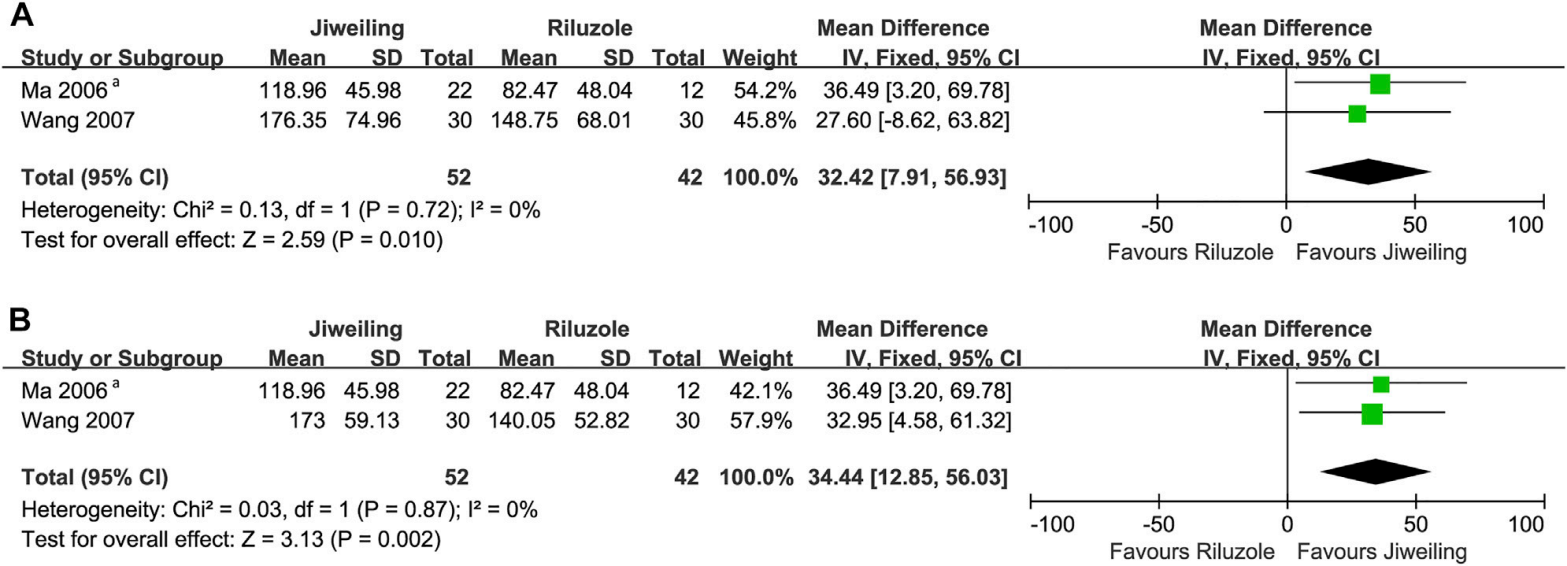
Effect of *Jiweiling* injection on MUNE in patients with amyotrophic lateral sclerosis. **(A)** The MUNE detected from the right abductor pollicis brevis in Wang 2007 was used. **(B)** The MUNE detected from the left abductor pollicis brevis in Wang 2007 was used. *MUNE*, motor unit number estimation. ^a^Which side of abductor pollicis brevis was tested was no noted in Ma 2006.

The MUNE detected from the right and left abductor pollicis brevis (APB) were pooled respectively in our analysis to reduce the heterogeneity, and the results favoured *Jiweiling* injection (right APB: MD, 32.42; 7.91 to 56.93; *p* = 0.01; left APB: MD, 34.44; 12.85 to 56.03; *p* = 0.002) compared to Riluzole (Figure 6). No subgroup or sensitivity analysis was conducted due to the insufficient number of trials.

Two trials (Supplementary Appendix A4) evaluated motor neuron loss using the amplitude of compound muscle action potential (Su et al., 2006; Wang et al., 2009), and they did not show better effects than controls.

Another trial (*N* = 78, Supplementary Appendix A4) used other electrophysiologic parameters related to muscle denervation and showed no statistical significance (Li, 2019b).

#### 3.7.8 Pharmacodynamic biomarkers

Three trials (*N* = 221, Supplementary Appendix A4) measured five types of biofluid markers (a total of fourteen biomarkers): creatine kinase, oxidative stress biomarkers, neuron-specific enolase, amino acid, and immunoglobulin (Wang, 2007; Wang et al., 2009; Chico et al., 2018). The corresponding results of comparisons between treatments and controls are presented in Supplementary Appendix A4, and two trials reported significant differences (Wang, 2007; Chico et al., 2018). Unfortunately, no trials measured neurofilament, the most promising candidate biomarker at present.

### 3.8 Adverse events

A total of 14 studies (*N* = 845) evaluated the safety of treatments (Table 1). Of these, nine studies reported the occurrence of 123 adverse events (Table 2). 26 AEs happened in the treatment groups and 97 in the control groups. Of the 26 AEs, 2 SAEs (death) occurred in two trials that investigated *Jiawei Sijunzi* formula and *Jianpi Yifei* formula, respectively (Pan et al., 2013a; Fang, 2016). Two participants receiving *Jianpi Yifei* formula added on Riluzole were reported abnormal liver function (Wang et al., 2017). Gastrointestinal discomforts were found in a total of 17 individuals who were treated with Cannabinoids (*N* = 5) (Riva et al., 2019), Curcumin (*N* = 4) (Chico et al., 2018), *Huoling Shengji* formula (*N* = 3) (Sui et al., 2016), *Jianpi Yifei* formula plus Riluzole (*N* = 1) (Wang et al., 2017), and *Jiawei Sijunzi* formula (*N* = 4) (Pan et al., 2013a), respectively. Three participants taking *Jianpi Yifei* formula plus Riluzole developed skin allergies (Wang et al., 2017), and one patient receiving Curcumin had a skin rash (Chico et al., 2018). In addition, one patient who took Cannabinoids was reported cardiovascular disease.

**TABLE 2:** Occurrence of adverse events in randomised clinical trials of herbal medicine for amyotrophic lateral sclerosis.

Study	Treatment	Control	Treatment total *N* = 265	Control total *N* = 269	Treatment AEs *N* = 26	Control AEs *N* = 97
Chico et al. (2018)	Curcumin	placebo	18	24	Gastrointestinal disorder (4)	Cardiovascular disease (1)
					Skin disorder (1)	
Fang (2016)	*Jianpi Yifei* formula	Riluzole	30	30	Death (1)^a^	Death (2)^a^
Ma (2006)	*Jiweiling* injection + dummy Riluzole	Riluzole + dummy *Jiweiling* injection	30	30	0	Hepatic injury (22)
Pan et al. (2013a)	*Jiawei Sijunzi* formula	Riluzole	24	24	Death (1)^a^	Death (2)^a^
					Gastrointestinal disorder (4)	Gastrointestinal disorder (12)
Pan (2015)	*Shenzhe Jiangqi* powder + dummy Riluzole	Riluzole + dummy *Shenzhe Jiangqi* powder	40	40	0	Hepatic injury (13)
						Gastrointestinal disorder (5)
Riva et al. (2019)	Cannabinoids	placebo	30	30	Gastrointestinal disorder (5)	Gastrointestinal disorder (2)
					Cardiovascular disease (1)	Skin disorder (3)
Sui et al. (2016)	*Huoling Shengji* formula	Riluzole	33	31	Gastrointestinal disorder (3)	Gastrointestinal disorder (16)
Wang (2007)	*Jiweiling* injection	Riluzole	30	30	0	Hepatic injury (13)
Wang 2017a	*Jianpi Yifei* formula + Riluzole	Riluzole	30	30	Hepatic injury (2)	Hepatic injury (1)
					Gastrointestinal disorder (1)	Gastrointestinal disorder (1)
					Skin disorder (3)	Skin disorder (4)

a Serious adverse events.

### 3.9 Certainty of evidence

All outcome measurements for evaluation of function, survival, biofluid markers, and electrophysiological markers were rated using GRADE. All included evidence was very low to low quality (Table 3). The risk of bias and imprecision were the reasons for downgrading all outcomes.

**TABLE 3:** GRADE evidence profile: herbal medicine for patients with amyotrophic lateral sclerosis.

Outcomes	Measures	Participants, studies	Risk of bias	Inconsistency	Indirectness	Imprecision	Publication bias	Certainty	Effect estimate^a^	
									Control	Herbal medicine
Activity limitation	ALSFRS-R/ALSFRS	721, 14	Serious^b^	Not serious	Not serious	Serious limitations^c^	Undetected	Low	25.38	SMD 0.64 more (0.47–0.80 more)
	Modified Norris Scale	438, 7	Serious^b^	Not serious	Not serious	Serious limitations^c^	Undetected	Low	58.22	MD 0.60 more (0.33–0.86 more)
Survival		58, 1	Serious^b^	Not serious	Not serious	Serious limitations^c^	Undetected	Low	n/N, 20/28	Not significant, RR 1.12 (0.83–1.50)^d^
Loss of strength	FVC	291, 5	Serious^b^	Serious^e^	Not serious	Serious limitations^c^	Undetected	Very low	69.95%	MD 4.46% more (1.08–7.84% more)
	VC	120, 2	Serious^b^	Serious^e^	Not serious	Serious limitations^c^	Undetected	Very low	68.08%	MD 5.35% more (2.34–8.36% more)
	MRC	191, 4	Serious^b^	Not serious	Not serious	Serious limitations^c^	Undetected	Low	5.76	Not significant, MD 0.04 more (0.28 fewer to 0.36 more)
	HHD	36, 1	Serious^b^	Not serious	Not serious	Serious limitations^c^	Undetected	Low	NA	NA
Quality of life	ALSAQ-40	233, 4	Serious^b^	Not serious	Not serious	Serious limitations^c^	Undetected	Low	56.88	ND^f^
	SF-36 (sub)	42, 1	Serious^b^	Not serious	Not serious	Serious limitations^c^	Undetected	Low	37.60	Not significant, MD 0.80 more (3.20 fewer to 4.80 more)
	Barthel index	119, 2	Serious^b^	Serious^e^	Not serious	Serious limitations^c^	Undetected	Very low	86.16	MD 3.67 more (0.06–7.28 more)
Functional status	AALSS	138, 2	Serious^b^	Not serious	Not serious	Serious limitations^c^	Undetected	Low	84.02	MD 5.47 fewer (9.34–1.60 fewer)
Traditional medicine syndrome		560, 9	Serious^b^	Serious^e^	Serious^g^	Serious limitations^c^	Undetected	Very low	15.29	SMD 0.81 fewer (1.04–0.58 fewer)
Motor neuron loss	MUNE	230, 4	Serious^b^	Serious^e^	Not serious	Serious limitations^c^	Undetected	Very low	104.56	MD 34.75 more (18.74–50.76 more)
	CMAP	35^h^, 2	Serious^b^	Not serious	Not serious	Serious limitations^c^	Undetected	Low	53.57	Not significant, MD 1.00 more (0.84 fewer to 2.84 more)
Pharmacodynamic biomarkers		221, 3	Serious^b^	Serious^e^	Not serious	Serious limitations^c^	Undetected	Very low	34.42	ND^i^
Adverse events		845, 14	Serious^b^	Serious^e^	Not serious	Serious limitations^c^	Undetected	Very low	n/N, 97/269	RR 0.28 (0.19–0.42)

AALSS, appel amyotrophic lateral sclerosis score; ALSAQ-40, amyotrophic lateral sclerosis assessment questionnaire-40; ALSFRS, amyotrophic lateral sclerosis functional rating scale; ALSFRS-R, amyotrophic lateral sclerosis functional rating scale-revised; CMAP, compound muscle action potential; FVC, forced vital capacity; GRADE, the grading of recommendations assessment, development, and evaluation; HHD, hand-held dynamometry; MRC, medical research council scale; MUNE, motor unit number estimation; NA, not available; ND, not done; SF-36, MOS item short-form health survey-36; VC, vital capacity.

a We chose to combine studies with post intervention values here, and studies with changes from baseline were listed in Supplementary Appendix A4.

b Hiding or binding was not used.

c Small number of events, or confidence interval was too wide.

d O-E and variance were not available.

e Point estimates varied widely across studies, confidence intervals showed minimal, the heterogeneity test was significant, or the I^2^ was large.

f The trials misunderstood the clinical meaning of the ALSAQ-40, thus the data synthesis was abandoned.

g Lack of consistent and objective diagnostic criteria.

h The number of participants in one of trials was unclear.

i Whether all the decreases of biomarkers point to the same direction is uncertain.

## 4 Discussion

### 4.1 Summary of findings

Twenty studies of HM for ALS were included. Whether these clinical trials are futile under the circumstances that so many efforts have been made but the effects of HMs are still being questioned. We systematically appraised published RCTs of HM for ALS to address this issue. Our results suggest that HMs may be effective for improvement in activity limitation, muscle strengths, QOL, functional status, traditional medicine syndromes, and motor neuron loss for individuals with ALS. However, only low and very low quality of evidence was available, which restricts the confidence that can be placed in the findings. Results of the meta-analysis revealed significant improvement in activity limitation (*Jiweiling* injection and *Shenmai* injection) and motor neuron loss (*Jiweiling* injection) when patients were treated with finished herbal productions. Nevertheless, the less number of trials brought into analysis reduce the reliability of the results to some extent. In addition, there was insufficient evidence of HMs prolonging survival.

Of these included herbal medicines, several possible therapeutic mechanisms were reported. Antioxidant was associated with the neuroprotective actions of ALS in *Shenmai* injection (She et al., 2013), Curcumin (Zhang et al., 2014), *Guilu Erxian* glues (Xv et al., 2013), *Huoling Shengji* formula (Zhou, 2017), and *Jianpi Yifei* formula (She et al., 2013; Li, 2019). In addition, *Jiweiling* injection plays a role in inhibiting calcium toxicity and anti-apoptosis (Wang, 2005). Cannabinoids exert effects on anti-inflammatory and antioxidant (Alexander, 2016). The pharmacologic mechanisms of the other seven HMs lack reports. However, *Astragalus mongholicus Bunge*, with properties of anti-aggregation of proteins and anti-inflammation (Zhang et al., 2014), is the main herb of four of them (*Bushen Jianpi Shugan* formula, *Fuyuan Shengji* granule, *Jiawei Sijunzi* formula, and *Yiqi Qiangji* formula). And ginseng, which has a neuroprotective effect against neuroinflammation and oxidative stress (Cai and Yang, 2016), is the main herb of *Shenzhe Jiangqi* powder.

When faced with significant evidence with very low to low quality, critical analyses may benefit the future clinical trials of HM for ALS. In the context of disease heterogeneity in ALS, the restrictive inclusion criteria for phase Ⅱ and Ⅲ clinical trials are becoming an important consideration (Kiernan et al., 2021). The stratification of patients at the time of recruitment according to their characteristics enables patients to be matched with suitable therapies. Our included HM studies were mainly small sample clinical trials and thus should have a higher requirement of recruiting homogeneous subjects. Instead, participants with little restriction on diagnostic certainties and disease course were recruited and treated without stratification. Nowadays, several prognostic models and tools have been proposed to optimize ALS trials, especially in the recruitment process (Westeneng et al., 2018; van Eijk et al., 2019; van Eijk et al., 2021). Under the circumstances that ALS clinical trials with large sample sizes are difficult to be carried out, employing these models to select appropriate populations of patients sensitive to HMs makes sense.

Concerning the treatments, some trials did not describe the source and concentration of herbal reparations (Supplementary Appendix A3). Moreover, our meta-analysis showed statistical significance when data from finished herbal products were used. These findings suggest that unspecific constituents and manufacturing processes may hinder objective HM efficacy evaluations and thus reduce the repeatability of HM tested in further clinical trials. In addition, most HM trials only investigated patients for 3 months or less (Table 1). Such a short duration is not long enough to provide confirmatory evidence.

Regarding the comparators, in order to reduce unnecessary exposure to placebos, eight studies compared investigated HMs with Riluzole. However, such a design hampers the recognition of HM’s net effect. The master protocol (Kiernan et al., 2021), a strategy aiming to minimize unnecessary exposure to placebo and allowing for the simultaneous evaluation of multiple treatments with a shared placebo group, is a promising approach to address this issue. In addition, the Food and Drug Administration recommends the consideration of add-on designs in ALS clinical trials (The Food and Drug Administration, 2019), which is another choice to comply with the ethical criteria.

In terms of the selection of outcome measures, some limitations were identified. First, the ALSFRS-R/ALSFRS is the most widely accepted outcome measure of activity limitation in ALS patients (Gordon et al., 2007; Cudkowicz et al., 2014; Abe et al., 2017; Paganoni et al., 2020). However, about one-fourth of the included trials measuring activity limitation did not employ them but used the modified Norris Scale instead, which is less favourable at present. Furthermore, most HM clinical trials did not measure survival in consideration of disease heterogeneity, variation in the expected disease course, and high cost due to extended follow-up. However, the comparison of the design of clinical trials between Riluzole (Bensimon et al., 1994; Lacomblez et al., 1996) and Edaravone (Abe et al., 2017) shows that an increased life expectancy leaves little doubt about a treatment’s therapeutic potential. Thus, the benefits of measuring survival time in HM clinical trials may outweigh the disadvantages. Additionally, incorporation of optimized biomarkers into early-stage clinical trials is in prospect. Inspiringly, some of the included studies measured biofluid markers or electrophysiological markers and even drew significant conclusions, which benefits the understanding of pharmacological mechanisms. Rapid advances in the detection of the molecular biology and pathology of ALS are making the novel biomarker constantly emerge but also leading to the phenomenon that various biomarkers are employed without a uniform standard. Meanwhile, reliable biomarkers are badly needed for monitoring the response to treatment, but the consensus about the robust candidates has not yet been established, even though some high-quality publications have made recommendations (Grossman et al., 2014; Benatar et al., 2018; Magen et al., 2021), and certain biomarkers, such as the neurofilament light chain, have been already used in multicentre clinical trials (Paganoni et al., 2020). In addition, it is found that two studies (Ma, 2006; Wang, 2007) misunderstood the clinical meaning of the ALSAQ-40. Therefore, we abandoned the relevant data synthesis, even if they were homogenous and claimed to be effective.

Furthermore, other flaws in methodology could also hinder the recognition of the efficacy of HMs. Fifteen studies employed an open-labelled design because of common difficulties in imitating the smell and appearance of dummy herbal products, especially the peroral dosage form, which increased the information bias. Notably, most included studies claimed to be RCTs. However, the investigators did not sufficiently describe details related to the sample size calculation, randomization process, implementation of blinding, or measurement outcomes. Hence, reporting in detail according to the Consolidated Standards of Reporting Trials (CONSORT) Statement is urgently needed. Additionally, one study used a 10th of the dose of the investigated medicine as the placebo. Such a methodological flaw dramatically reduced the reliability of the study’s evidence.

Some inherent limitations in HM clinical trials mentioned above are hard to be remedied in the short run. A registration study with heterogeneous populations and long-term follow-up can bring researchers and patients objective and comprehensive knowledge about disease trajectory and even effectiveness of HMs via statistical methods. Such a study has been established in mainland China to investigate the properties of ALS patients who take HM (CARE-TCM) (Song et al., 2022). In addition, we had noticed that a new HM called TJ-68 (*Shaoyao Gancao* formula) was expected to be tested in an N-of-1 study and the RCT of *Huoling Shengji* formula was on-going while this manuscript was drafted. More evidence derived from well-designed trials may update our understanding of HM for ALS.

Some evidence shows more extensive involvement of pathological changes in ALS than previously recognized, such as cognitive dysfunction, emotional instability, and insomnia (Sedda, 2014; Boentert, 2020; Pender et al., 2020). It is found that these symptoms related to the extra-motor system are not rare concomitant behaviours and directly impact QOL even though they hardly threaten survival. This systematic review aimed to appraise the effects of HMs on motor system symptoms. Further reviews summarizing the therapeutic effects of HMs for extra-motor system symptoms are needed.

Nine of twenty studies reported AEs, and two reported SAEs (Table 2). The occurrences of AEs, such as liver dysfunction, and SAEs, such as death, were more frequent in the control groups.

### 4.2 Limitations

This systematic review had some limitations. First, a large number of potential descriptors for HM hinder the design of a search strategy, even though we have used the Cochrane sensitivity-maximizing filter for RCTs to highly identify clinical trials. Second, the less number of trials with a small sample size included in the meta-analysis reduces the reliability of the pooled results. Third, the lack of data from on-going trials may alter the results dramatically.

## 5 Conclusion

HMs may play a role in delaying decline in function, and the evidence for the role in extending survival was insufficient. The very low to low quality of evidence requires further RCTs that have adequate methods, use placebos as controls, select appropriate participants, and employ efficient outcome measures.

## 6 Amendments to information provided at registration

We reviewed the results of measurements based on traditional medicine theory, which was not stated at registration.

## Data Availability

The original contributions presented in the study are included in the article/Supplementary Material, further inquiries can be directed to the corresponding authors.

## References

[B1] Abe K. Aoki M. Tsuji S. Itoyama Y. Sobue G. Togo M. (2017). Safety and efficacy of edaravone in well defined patients with amyotrophic lateral sclerosis: A randomised, double-blind, placebo-controlled trial. Lancet Neurol. 16 (7), 505–512. 10.1016/s1474-4422(17)30115-1 PubMed Abstract | 10.1016/s1474-4422(17)30115-1 | Google Scholar 28522181

[B2] Alexander S. P. (2016). Therapeutic potential of cannabis-related drugs. Prog. Neuropsychopharmacol. Biol. Psychiatry 64, 157–166. 10.1016/j.pnpbp.2015.07.001 PubMed Abstract | 10.1016/j.pnpbp.2015.07.001 | Google Scholar 26216862

[B3] Appel V. Stewart S. S. Smith G. Appel S. H. (1987). A rating scale for amyotrophic lateral sclerosis: Description and preliminary experience. Ann. Neurol. 22 (3), 328–333. 10.1002/ana.410220308 PubMed Abstract | 10.1002/ana.410220308 | Google Scholar 3118763

[B4] Bao J. Fu M. Shen B. Zhu X. (2016). Treatment of amyotrophic lateral sclerosis with Jiawei Sijunzi decoction. Her. Med. 35 (S1), 43–45. 10.3870/j.issn.1004-0781.2016.z1.021 10.3870/j.issn.1004-0781.2016.z1.021 | Google Scholar

[B5] Benatar M. Wuu J. Andersen P. M. Lombardi V. Malaspina A. (2018). Neurofilament light: A candidate biomarker of presymptomatic amyotrophic lateral sclerosis and phenoconversion. Ann. Neurol. 84 (1), 130–139. 10.1002/ana.25276 PubMed Abstract | 10.1002/ana.25276 | Google Scholar 30014505 PMC11348288

[B6] Bensimon G. Lacomblez L. Meininger V. (1994). A controlled trial of riluzole in amyotrophic lateral sclerosis. ALS/Riluzole Study Group. N. Engl. J. Med. 330 (9), 585–591. 10.1056/NEJM199403033300901 PubMed Abstract | 10.1056/NEJM199403033300901 | Google Scholar 8302340

[B7] Boentert M. (2020). Sleep and sleep disruption in amyotrophic lateral sclerosis. Curr. Neurol. Neurosci. Rep. 20 (7), 25. 10.1007/s11910-020-01047-1 PubMed Abstract | 10.1007/s11910-020-01047-1 | Google Scholar 32462331 PMC7253511

[B8] Brooks B. R. (1994). El escorial World federation of neurology criteria for the diagnosis of amyotrophic lateral sclerosis. Subcommittee on motor neuron diseases/amyotrophic lateral sclerosis of the World federation of neurology research group on neuromuscular diseases and the El escorial "clinical limits of amyotrophic lateral sclerosis" workshop contributors. J. Neurol. Sci. 124, 96–107. 10.1016/0022-510x(94)90191-0 PubMed Abstract | 10.1016/0022-510x(94)90191-0 | Google Scholar 7807156

[B9] Brooks B. R. Miller R. G. Swash M. Munsat T. L. (2000). El escorial revisited: Revised criteria for the diagnosis of amyotrophic lateral sclerosis. Amyotroph. Lateral Scler. Other Mot. Neuron Disord. 1 (5), 293–299. 10.1080/146608200300079536 PubMed Abstract | 10.1080/146608200300079536 | Google Scholar 11464847

[B10] Brown R. H. Al-Chalabi A. (2017). Amyotrophic lateral sclerosis. N. Engl. J. Med. 377 (2), 162–172. 10.1056/NEJMra1603471 PubMed Abstract | 10.1056/NEJMra1603471 | Google Scholar 28700839

[B11] Cai M. Lee S. H. Yang E. J. (2019). Bojungikgi-tang improves muscle and spinal cord function in an amyotrophic lateral sclerosis model. Mol. Neurobiol. 56 (4), 2394–2407. 10.1007/s12035-018-1236-0 PubMed Abstract | 10.1007/s12035-018-1236-0 | Google Scholar 30030751

[B12] Cai M. Yang E. J. (2016). Ginsenoside Re attenuates neuroinflammation in a symptomatic ALS animal model. Am. J. Chin. Med. 44 (2), 401–413. 10.1142/s0192415x16500233 PubMed Abstract | 10.1142/s0192415x16500233 | Google Scholar 27080948

[B13] Cai R. (2011). Shenmai injection combined with comprehensive therapy for motor neuron disease: A clinical review of 38 cases. J. Math. Med. 24 (05), 534–535. Google Scholar

[B14] Cedarbaum J. M. Stambler N. Malta E. Fuller C. Hilt D. Thurmond B. (1999). The ALSFRS-R: A revised ALS functional rating scale that incorporates assessments of respiratory function. BDNF ALS study group (phase III). J. Neurol. Sci. 169 (1-2), 13–21. 10.1016/s0022-510x(99)00210-5 PubMed Abstract | 10.1016/s0022-510x(99)00210-5 | Google Scholar 10540002

[B15] Chico L. Ienco E. C. Bisordi C. Lo Gerfo A. Petrozzi L. Petrucci M. (2018). Amyotrophic lateral sclerosis and oxidative stress: A double-blind therapeutic trial after curcumin supplementation. CNS Neurol. Disord. Drug Targets 17 (10), 767–779. 10.2174/1871527317666180720162029 PubMed Abstract | 10.2174/1871527317666180720162029 | Google Scholar 30033879

[B16] Cudkowicz M. E. Titus S. Kearney M. Yu H. Sherman A. Schoenfeld D. (2014). Safety and efficacy of ceftriaxone for amyotrophic lateral sclerosis: A multi-stage, randomised, double-blind, placebo-controlled trial. Lancet. Neurol. 13 (11), 1083–1091. 10.1016/S1474-4422(14)70222-4 PubMed Abstract | 10.1016/S1474-4422(14)70222-4 | Google Scholar 25297012 PMC4216315

[B17] de Carvalho M. Dengler R. Eisen A. England J. D. Kaji R. Kimura J. (2008). Electrodiagnostic criteria for diagnosis of ALS. Clin. Neurophysiol. 119 (3), 497–503. 10.1016/j.clinph.2007.09.143 PubMed Abstract | 10.1016/j.clinph.2007.09.143 | Google Scholar 18164242

[B18] Fang Z. (2016). Master. Guangzhou: Guangzhou University of Chinese Medicine. Assessment of bulbar palsy in amyotrophic lateral sclerosis patients and evaluation of the effect of Jianpiyifei Decoction Google Scholar

[B19] Gordon P. H. Moore D. H. Miller R. G. Florence J. M. Verheijde J. L. Doorish C. (2007). Efficacy of minocycline in patients with amyotrophic lateral sclerosis: A phase III randomised trial. Lancet. Neurol. 6 (12), 1045–1053. 10.1016/S1474-4422(07)70270-3 PubMed Abstract | 10.1016/S1474-4422(07)70270-3 | Google Scholar 17980667

[B20] Grossman M. Elman L. McCluskey L. McMillan C. T. Boller A. Powers J. (2014). Phosphorylated tau as a candidate biomarker for amyotrophic lateral sclerosis. JAMA Neurol. 71 (4), 442–448. 10.1001/jamaneurol.2013.6064 PubMed Abstract | 10.1001/jamaneurol.2013.6064 | Google Scholar 24492862 PMC3989393

[B21] Jenkinson R. Fitzpatrick B. Brennan C. SwashM. M. (1999). Evidence for the validity and reliability of the ALS assessment questionnaire: The ALSAQ-40. Amyotroph. Lateral Scler. Other Mot. Neuron Disord. 1 (1), 33–40. 10.1080/146608299300080022 PubMed Abstract | 10.1080/146608299300080022 | Google Scholar 12365067

[B22] Jin P. (2013). Clinical observation on treating amyotrophic lateral sclerosis by Guilu Erxian glues. Clin. J. Chin. Med. 5 (24), 28–30. 10.3969/j.issn.1674-7860.2013.24.012 10.3969/j.issn.1674-7860.2013.24.012 | Google Scholar

[B23] Kiernan M. C. Vucic S. Talbot K. McDermott C. J. Hardiman O. Shefner J. M. (2021). Improving clinical trial outcomes in amyotrophic lateral sclerosis. Nat. Rev. Neurol. 17 (2), 104–118. 10.1038/s41582-020-00434-z PubMed Abstract | 10.1038/s41582-020-00434-z | Google Scholar 33340024 PMC7747476

[B24] Lacomblez L. Bensimon G. Leigh V. Guillet P. N. Meininger P. (1996). Dose-ranging study of riluzole in amyotrophic lateral sclerosis. Amyotrophic Lateral Sclerosis/Riluzole Study Group II. Lancet 347 (9013), 1425–1431. 10.1016/S0140-6736(96)91680-3 PubMed Abstract | 10.1016/S0140-6736(96)91680-3 | Google Scholar 8676624

[B25] Lefebvre C. Glanville J. Briscoe S. Littlewood A. Marshall C. Metzendorf M-I. (2021). Cochrane Handbook for systematic reviews of interventions.Chapter 4: Searching for and selecting studies Google Scholar

[B26] Li C. Hu L. Kong L. Gao J. Zhu X. Zhi H. (2011). Efficacy of Fuyuanshengji Granule on the short period prognosis of the patients with amyotrophic lateral sclerosis. J. Neurology Neurorehabilitation 8 (02), 61–64. Google Scholar

[B27] Li H. Z. (2019a). Effect of Jianpi Yifei Formula on glutamate induced spinal cord neuron injury. Guangzhou: Master, Guangzhou University of traditional Chinese Medicine. Google Scholar

[B28] Li P. (2019b). Effects of Jianpi Yifei formula combined with massage on neurological function and electromyography in patients with amyotrophic lateral sclerosis. J. Sichuan Traditional Chin. Med. 37 (8), 147–149. Google Scholar

[B29] Ma W. (2006). Master. Shijiazhuang: Hebei Medical University.The clinical study of Jiweiling injection in dealing with amyotrophic lateral sclerosis Google Scholar

[B30] Magen I. Yacovzada N. S. Yanowski E. Coenen-Stass A. Grosskreutz J. Lu C. H. (2021). Circulating miR-181 is a prognostic biomarker for amyotrophic lateral sclerosis. Nat. Neurosci. 24 (11), 1534–1541. 10.1038/s41593-021-00936-z PubMed Abstract | 10.1038/s41593-021-00936-z | Google Scholar 34711961

[B31] Norris F. H. Jr. Calanchini P. R. Fallat R. J. Panchari S. Jewett B. (1974). The administration of guanidine in amyotrophic lateral sclerosis. Neurology 24 (8), 721–728. 10.1212/wnl.24.8.721 PubMed Abstract | 10.1212/wnl.24.8.721 | Google Scholar 4858705

[B32] Paganoni S. Cudkowicz M. Berry J. D. (2014). Outcome measures in amyotrophic lateral sclerosis clinical trials. Clin. Investig. (Lond) 4 (7), 605–618. 10.4155/cli.14.52 PubMed Abstract | 10.4155/cli.14.52 | Google Scholar PMC5305182 28203356

[B33] Paganoni S. Macklin E. A. Hendrix S. Berry J. D. Elliott M. A. Maiser S. (2020). Trial of sodium phenylbutyrate-taurursodiol for amyotrophic lateral sclerosis. N. Engl. J. Med. 383 (10), 919–930. 10.1056/NEJMoa1916945 PubMed Abstract | 10.1056/NEJMoa1916945 | Google Scholar 32877582 PMC9134321

[B34] Page M. J. McKenzie J. E. Bossuyt P. M. Boutron I. Hoffmann T. C. Mulrow C. D. (2021). The PRISMA 2020 statement: An updated guideline for reporting systematic reviews. BMJ 372, n71. 10.1136/bmj.n71 PubMed Abstract | 10.1136/bmj.n71 | Google Scholar 33782057 PMC8005924

[B35] Pan C. (2015). Clinical study on treatment of Chong meridian qi adversely ascending type amyotrophic lateral sclerosis by Shenzhe Jiangqi Powder Master. Shijiazhuang: Hebei Medical Univeristy. Google Scholar

[B36] Pan W. Chen X. Bao J. Bai Y. Lu H. Wang Q. (2013b). The use of integrative therapies in patients with amyotrophic lateral sclerosis in shanghai, China. Evid. Based. Complement. Altern. Med. 2013, 613596. 10.1155/2013/613596 PubMed Abstract | 10.1155/2013/613596 | Google Scholar PMC3865630 24363770

[B37] Pan W. Su J. Bao J. Wang J. Zhu D. Cai X. (2013a). Open randomized clinical trial on JWSJZ Decoction for the treatment of ALS patients. Evid. Based. Complement. Altern. Med. 2013, 347525. 10.1155/2013/347525 PubMed Abstract | 10.1155/2013/347525 | Google Scholar PMC3777196 24093046

[B38] Pender N. Pinto-Grau M. Hardiman O. (2020). Cognitive and behavioural impairment in amyotrophic lateral sclerosis. Curr. Opin. Neurol. 33 (5), 649–654. 10.1097/wco.0000000000000862 PubMed Abstract | 10.1097/wco.0000000000000862 | Google Scholar 32833751

[B39] Riva N. Mora G. Sorarù G. Lunetta C. Ferraro O. E. Falzone Y. (2019). Safety and efficacy of nabiximols on spasticity symptoms in patients with motor neuron disease (CANALS): A multicentre, double-blind, randomised, placebo-controlled, phase 2 trial. Lancet. Neurol. 18 (2), 155–164. 10.1016/s1474-4422(18)30406-x PubMed Abstract | 10.1016/s1474-4422(18)30406-x | Google Scholar 30554828

[B40] Sedda A. (2014). Disorders of emotional processing in amyotrophic lateral sclerosis. Curr. Opin. Neurol. 27 (6), 659–665. 10.1097/wco.0000000000000147 PubMed Abstract | 10.1097/wco.0000000000000147 | Google Scholar 25333604

[B41] She J. Xin Y. F. Xuan Y. X. (2013). Research Progress on the material basis of pharmacological action of Shenmai Injection. Her. Med. 32 (04), 497–500. Google Scholar

[B42] Shefner J. M. Al-Chalabi A. Baker M. R. Cui L. Y. de Carvalho M. Eisen A. (2020). A proposal for new diagnostic criteria for ALS. Clin. Neurophysiol. 131 (8), 1975–1978. 10.1016/j.clinph.2020.04.005 PubMed Abstract | 10.1016/j.clinph.2020.04.005 | Google Scholar 32387049

[B43] Simmons Z. Felgoise S. H. Bremer B. A. Walsh S. M. Hufford D. J. Bromberg M. B. (2006). The ALSSQOL: Balancing physical and nonphysical factors in assessing quality of life in ALS. Neurology 67 (9), 1659–1664. 10.1212/01.wnl.0000242887.79115.19 PubMed Abstract | 10.1212/01.wnl.0000242887.79115.19 | Google Scholar 17101900

[B44] Song Y. Li M. Sugimoto K. Han Y. Liu J. Ma B. (2022). China amyotrophic lateral sclerosis registry of patients with Traditional Chinese Medicine (CARE-TCM): Rationale and design. J. Ethnopharmacol. 284, 114774. 10.1016/j.jep.2021.114774 PubMed Abstract | 10.1016/j.jep.2021.114774 | Google Scholar 34699945

[B45] Sterne J. A. C. Savović J. Page M. J. Elbers R. G. Blencowe N. S. Boutron I. (2019). RoB 2: A revised tool for assessing risk of bias in randomised trials. BMJ 366, l4898. 10.1136/bmj.l4898 PubMed Abstract | 10.1136/bmj.l4898 | Google Scholar 31462531

[B46] Su G. Zhang J. Hong Y. (2006). Treatment of 25 cases of amyotrophic lateral sclerosis with Yiqi Qiangji Decoction. Chin. J. Integr. Med. Cardio-Cerebrovascular Dis. 4 (05), 452–453. Google Scholar

[B47] Sugimoto K. Liu J. Li M. Song Y. Zhang C. Zhai Z. (2021). Neuroprotective effects of Shenqi Fuzheng injection in a transgenic SOD1-g93a mouse model of amyotrophic lateral sclerosis. Front. Pharmacol. 12, 701886. 10.3389/fphar.2021.701886 PubMed Abstract | 10.3389/fphar.2021.701886 | Google Scholar 34737697 PMC8560685

[B48] Sui S. Wang Y. Zhi H. Hong Y. Feng Y. (2016). Clinical observation of Huoling Shengji formula in the treatment of amyotrophic lateral sclerosis. Acad. J. Shanghai Univ. Traditional Chin. Med. 30 (02), 23–26. 10.16306/j.1008-861x.2016.02.006 10.16306/j.1008-861x.2016.02.006 | Google Scholar

[B49] The ALS CNTF treatment study (ACTS) phase I-II Study Group (1996). The Amyotrophic Lateral Sclerosis Functional Rating Scale. Assessment of activities of daily living in patients with amyotrophic lateral sclerosis. The ALS CNTF treatment study (ACTS) phase I-II Study Group. Arch. Neurol. 53 (2), 141–147. 10.1001/archneur.1996.00550020045014 PubMed Abstract | 10.1001/archneur.1996.00550020045014 | Google Scholar 8639063

[B50] The Food and Drug Administration (2019). Amyotrophic lateral sclerosis-developing drugs for treatment guidance for industry. Available at: https://www.fda.gov/regulatory-information/search-fda-guidance-documents/amyotrophic-lateral-sclerosis-developing-drugs-treatment-guidance-industry . Google Scholar

[B51] The World Health Organization (2021). Traditional Chinese medicine could make "Health for one" true. Available at: https://www.who.int/intellectualproperty/studies/Jia.pdf?ua=1 . Google Scholar

[B52] van Eijk R. P. A. Nikolakopoulos S. Roes K. C. B. Kendall L. Han S. S. Lavrov A. (2021). Innovating clinical trials for amyotrophic lateral sclerosis: Challenging the established order. Neurology 97 (11), 528–536. 10.1212/WNL.0000000000012545 PubMed Abstract | 10.1212/WNL.0000000000012545 | Google Scholar 34315786 PMC8456357

[B53] van Eijk R. P. A. Nikolakopoulos S. Roes K. C. B. Middelkoop B. M. Ferguson T. A. Shaw P. J. (2019). Critical design considerations for time-to-event endpoints in amyotrophic lateral sclerosis clinical trials. J. Neurol. Neurosurg. Psychiatry 90 (12), 1331–1337. 10.1136/jnnp-2019-320998 PubMed Abstract | 10.1136/jnnp-2019-320998 | Google Scholar 31292200 PMC6902062

[B54] Vardeny O. Bromberg M. (2005). The use of herbal supplements and alternative therapies by patients with amyotrophic lateral sclerosis (ALS). J. Herb. Pharmacother. 5 (3), 23–31. 10.1080/j157v05n03_03 PubMed Abstract | 10.1080/j157v05n03_03 | Google Scholar 16520295

[B55] Wang A. Li X. Ren Z. Hou X. Lu M. Du B. (2017). Therapeutic effect of strengthening spleen and tonifying lung on amyotrophic lateral sclerosis. World Chin. Med. 12 (06), 1364–1367. PubMed Abstract | Google Scholar

[B56] Wang J. Gao J. Guo Y. Qin B. Ren H. Xv W. (2009). Clinical study of the effect of Fuyuan Shengji Granuleon symptoms of the patients with amyotrophic lateral sclerosis. J. Neurology Neurorehabilitation 6 (03), 173–175. Google Scholar

[B57] Wang J. M. (2005). Neuroprotective effect of Jiweiling injection on motor neuron disease. Doctor: Hebei Medical University. Google Scholar

[B58] Wang W. (2017). *Clinical observation on the treatment of motor neuron disease by nourishing kidney and strengthening spleen (liver-kidney yin deficiency type)* Master. Beijing: Henan University of Chinese Medicine. Google Scholar

[B59] Wang X. (2007). *The clinical study of Jiweiling injection in dealing with amyotrophic lateral sclerosis bulbar paralysis* Master. Shijiazhuang: Hebei Medical University. Google Scholar

[B60] Ware J. E. Jr. Sherbourne C. D. (1992). The MOS 36-item short-form health survey (SF-36). I. Conceptual framework and item selection. Med. Care 30 (6), 473–483. 10.1097/00005650-199206000-00002 PubMed Abstract | 10.1097/00005650-199206000-00002 | Google Scholar 1593914

[B61] Westeneng H.-J. Debray T. P. A. Visser A. E. van Eijk R. P. A. Rooney J. P. K. Calvo A. (2018). Prognosis for patients with amyotrophic lateral sclerosis: Development and validation of a personalised prediction model. Lancet. Neurol. 17 (5), 423–433. 10.1016/s1474-4422(18)30089-9 PubMed Abstract | 10.1016/s1474-4422(18)30089-9 | Google Scholar 29598923

[B62] Xv W. Ren H. Zhi H. (2011). Clinical observation on the treatment of amyotrophic lateral sclerosis by Tonifying the kidney, strengthening the spleen and soothing the liver. Acad. J. Shanghai Univ. Traditional Chin. Med. 25 (05), 46–49. 10.16306/j.1008-861x.2011.05.014 10.16306/j.1008-861x.2011.05.014 | Google Scholar

[B63] Xv Y. Zhong Y. Z. Lin S. Y. (2013). Experimental study and clinical application of Guilu Erxian glue. Heilongjiang J. Traditional Chin. Med. 42 (06), 72–73. Google Scholar

[B64] Zhang N. (2020). Effect of Shenmai Injection on patients with amyotrophic lateral sclerosis. Med. J. Chin. People's Health 32 (8), 49–50. 10.3969/j.issn.1672-0369.2020.08.019 10.3969/j.issn.1672-0369.2020.08.019 | Google Scholar

[B65] Zhang X. Hong Y. L. Xu D. S. Feng Y. Zhao L. J. Ruan K. F. (2014). A review of experimental research on herbal compounds in amyotrophic lateral sclerosis. Phytother. Res. 28 (1), 9–21. 10.1002/ptr.4960 PubMed Abstract | 10.1002/ptr.4960 | Google Scholar 23519768

[B66] Zhou Q. M. (2017). Neuroprotective therapy in amyotrophic lateral sclerosis model mice. Doctor: Shanghai Jiaotong University. Google Scholar

[B67] Zhou Q. Wang Y. Zhang J. Shao Y. Li S. Wang Y. (2018). Fingerprint analysis of Huolingshengji Formula and its neuroprotective effects in SOD1G93A mouse model of amyotrophic lateral sclerosis. Sci. Rep. 8 (1), 1668. 10.1038/s41598-018-19923-9 PubMed Abstract | 10.1038/s41598-018-19923-9 | Google Scholar 29374221 PMC5786035

[B68] Zhu X. (2016). Exploratory study on the action mechanism of supplementary Sijunzi Decoction in the treatment of amyotrophic lateral sclerosis. Doctor: Shanghai University of Chinese Medicine. Google Scholar

