# Supplementary material for: Herbal medicine for amyotrophic lateral sclerosis: A systematic review and meta-analysis
Source: Front Pharmacol. 2022 Aug 31;13:946548. doi: 10.3389/fphar.2022.946548 (PMC9473725; doi:10.3389/fphar.2022.946548)
Supplement: Supplementary file 1 [file DataSheet1.docx]

***Supplementary Material***

# Appendix 1. Search strategies

# Appendix 2. Characteristics of excluded studies.

# Appendix 3. The chemical and botanical characterisation of the involved preparation according to the “Four Pillars of Best Practice”.

# Appendix 4. Outcome data

# Appendix 5. PRISMA 2020 checklist.

# Appendix 6. PRISMA 2020 for abstracts checklist.

# Appendix 1. Search strategies

## Ovid MEDLINE(R) and Epub Ahead of Print, In-Process, In-Data-Review & Other Non-Indexed Citations, Daily and Versions <1946 to March 25, 2022>

ID Search

1 exp Motor Neuron Disease/ 31449

2 (moto$1 neuron$1 disease$1 or moto?neuron$1 disease).mp. 9902

3 ((Lou Gehrig$1 adj5 syndrome$1) or (Lou Gehrig$1 adj5 disease)).mp. 236

4 charcot disease.tw. 28

5 Amyotrophic Lateral Sclerosis.mp. 31200

6 or/1-5 42464

7 randomized controlled trial.pt. 562568

8 controlled clinical trial.pt. 94763

9 randomized.ab. 555112

10 placebo.ab. 226692

11 drug therapy.fs. 2462476

12 randomly.ab. 378679

13 trial.ab. 592565

14 groups.ab. 2327882

15 or/7-14 5299179

16 exp animals/ not humans.sh. 4978655

17 15 not 16 4611132

18 exp Phytotherapy/ 41533

19 exp Medicine, traditional/ 43460

20 exp Plants, Medicinal/th, tu [Therapy, Therapeutic Use] 751

21 exp Herbal Medicine/ 2393

22 exp Plant Preparations/tu [Therapeutic Use] 47705

23 exp Plant extracts/tu [Therapeutic Use] 41725

24 (phyto adj6 (drug* or pharmaceutical* or therap* or treatment* or medicin*)).tw,ot. 237

25 (natur* adj3 compound*).tw,ot. 22580

26 exp Medicine, Chinese Traditional/ 22072

27 exp Drugs, Chinese herbal/ 49292

28 (chines* adj6 traditional medicin*).tw,ot. 1490

29 (Chinese adj3 (medic* or herb* or drug* or formul* or plant* or prescri*)).tw,ot. 56161

30 exp Kampo medicine/ 724

31 (japan* adj6 traditional medicin*).tw,ot. 199

32 (japanese adj3 (medic* or herb* or drug* or formul* or plant* or prescri*)).tw,ot. 3813

33 exp Medicine, Korean Traditional/ 487

34 (korea* adj6 traditional medicin*).tw,ot. 318

35 (Korean adj3 (medic* or herb* or drug* or formul* or plant* or prescri*)).tw,ot. 2519

36 or/18-35 186778

37 6 and 17 and 36 60

## Embase <1974 to 2022 March 25>

ID Search

1 exp Motor Neuron Disease/ 52056

2 (moto$1 neuron$1 disease$1 or moto?neuron$1 disease).mp. 15754

3 ((Lou Gehrig$1 adj5 syndrome$1) or (Lou Gehrig$1 adj5 disease)).mp. 252

4 charcot disease.tw. 36

5 Amyotrophic Lateral Sclerosis.mp. 47889

6 or/1-5 58166

7 randomized controlled trial/ 701420

8 crossover-procedure/ 69806

9 double-blind procedure/ 193516

10 single-blind procedure/ 45633

11 (random$ or factorial$ or crossover$ or cross over$ or cross-over$ or placebo$ or (doubl$ adj blind$) or (singl$ adj blind$) or assign$ or allocat$ or volunteer$).tw. 2535953

12 or/7-11 2641840

13 human/ 23275323

14 12 and 13 2071844

15 nonhuman/ or human/ 28228612

16 12 not 15 347546

17 14 or 16 2419390

18 exp phytotherapy/ 17632

19 traditional medicine/ 30858

20 exp medicinal plant/ 268413

21 exp herbal medicine/ 25574

22 exp plant medicinal product/dt [Drug Therapy] 391077

23 exp plant extracts/dt [Drug Therapy] 62500

24 exp plant preparations/dt [Drug Therapy] 391077

25 exp herbaceous agent/ 56865

26 (phyto adj3 (drug* or pharmaceutical* or therap* or treatment* or medicin*)).tw,ot. 321

27 (natur* adj3 compound*).tw,ot. 27649

28 exp Chinese medicine/ 60686

29 exp Drugs, Chinese herbal/ 56865

30 (chines* adj6 traditional medicin*).tw,ot. 1929

31 (Chinese adj3 (medic* or herb* or drug* or formul* or plant* or prescri*)).tw,ot. 81429

32 exp Kampo medicine/ 382

33 (japan* adj6 traditional medicin*).tw,ot. 299

34 (japanese adj3 (medic* or herb* or drug* or formul* or plant* or prescri*)).tw,ot. 5946

35 exp Medicine, Korean Traditional/ 1172

36 (korea* adj6 traditional medicin*).tw,ot. 421

37 (Korean adj3 (medic* or herb* or drug* or formul* or plant* or prescri*)).tw,ot. 5634

38 or/18-37 769786

39 6 and 17 and 38 73

## Web of science

ID Search

#4 #3 AND #2 AND #1 31

#3 ALL=("random*") 2,166,707

#2 TS=(Phytotherapy) OR TS=(Traditional medicine) OR TS=(medicinal plant) OR TS=(herb*) OR TS=(plant extract) OR TS=(plant preparation) OR TS=(natural compound*) OR TS=(Traditional Chinese medicine) OR TS=(Chinese drug) OR TS=(Chinese formul*) OR TS=(Chinese prescri*) OR TS=(kampo medicine) OR TS=(chinese materia medica) OR TS=(japanese medicine) OR TS=(japanese drug) OR TS=(japanese formul*) OR TS=(japanese prescri*) OR TS=(korean medicine) OR TS=(korean drug) OR TS=(korean formul*) OR TS=(korean prescri*) 677,159

#1 TS=(motor neuron disease) OR TS=(amyotrophic lateral sclerosis) OR TS=(Lou Gehrig* disease) OR TS=(Lou Gehrig* syndrome) OR TS=(charcot disease) 63,716

## The Cochrane Central Register of Controlled Trials (CENTRAL) in the Cochrane Library

ID Search

#1 MeSH descriptor: [Motor Neuron Disease] explode all trees 744

#2 (moto* neuron* disease* or moto?neuron* disease) 1490

#3 "Amyotrophic Lateral Sclerosis" 1471

#4 ("Lou Gehrig*" and (disease* or syndrome*)) 31

#5 "charcot disease" 19

#6 #1 OR #2 OR #3 OR #4 OR #5 2563

#7 MeSH descriptor: [Phytotherapy] explode all trees 4265

#8 MeSH descriptor: [Medicine, Traditional] explode all trees 1573

#9 MeSH descriptor: [Plants, Medicinal] explode all trees 947

#10 MeSH descriptor: [Herbal Medicine] explode all trees 63

#11 MeSH descriptor: [Plant Preparations] explode all trees and with qualifier(s): [therapeutic use - TU] 6307

#12 MeSH descriptor: [Plant Extracts] explode all trees and with qualifier(s): [therapeutic use - TU] 5411

#13 MeSH descriptor: [Drugs, Chinese Herbal] explode all trees 3687

#14 (phyto in All Text near/6 drug* in All Text) 4

#15 (phyto in All Text near/6 therap* in All Text) 4

#16 (phyto in All Text near/6 treatment* in All Text) 5

#17 (phyto in All Text near/6 medicin* in All Text) 3

#18 (natur* in All Text near/3 compound* in All Text) 1

#19 MeSH descriptor: [Medicine, Chinese Traditional] explode all trees 1230

#20 (Chinese in All Text near/3 medic* in All Text) 45

#21 (Chinese in All Text near/3 herb* in All Text) 6

#22 (Chinese in All Text near/3 drug* in All Text) 6

#23 (Chinese in All Text near/3 formul* in All Text) 7

#24 (Chinese in All Text near/3 plant* in All Text) 5

#25 (Chinese in All Text near/3 prescri* in All Text) 5

#26 MeSH descriptor: [Medicine, Kampo] explode all trees 46

#27 (Japanese in All Text near/3 medic* in All Text) 11

#28 (Japanese in All Text near/3 herb* in All Text) 0

#29 (Japanese in All Text near/3 drug* in All Text) 0

#30 (Japanese in All Text near/3 formul* in All Text) 0

#31 (Japanese in All Text near/3 plant* in All Text) 0

#32 (Japanese in All Text near/3 prescri* in All Text) 1

#33 MeSH descriptor: [Medicine, Korean Traditional] explode all trees 33

#34 (Korean in All Text near/3 medic* in All Text) 5

#35 (Korean in All Text near/3 herb* in All Text) 1

#36 (Korean in All Text near/3 drug* in All Text) 1

#37 (Korean in All Text near/3 formul* in All Text) 1

#38 (Korean in All Text near/3 plant* in All Text) 1

#39 (Korean in All Text near/3 prescri* in All Text) 1

#40 #7 OR #8 OR #9 OR #10 OR #11 OR #12 OR #13 OR #14 OR #15 OR #16 OR #17 OR #18 OR #19 OR #20 OR #21 OR #22 OR #23 OR #24 OR #25 OR #26 OR #27 OR #28 OR #29 OR #30 OR #31 OR #32 OR #33 OR #34 OR #35 OR #36 OR #37 OR #38 OR #39 9671

#41 #6 AND #40 in Trials 3

## Korean Journal Database (KCL)

ID Search

#4 #3 AND #2 AND #1 1

#3 TS=(random*) 36,999

#2 TS=(Phytotherapy) OR TS=(Traditional medicine) OR TS=(medicinal plant) OR TS=(herb*) OR TS=(plant extract) OR TS=(plant preparation) OR TS=(natural compound*) OR TS=(korean medicine) OR TS=(korean drug) OR TS=(korean formul*) OR TS=(korean prescri*) 33,256

#1 TS=(motor neuron disease) OR TS=(amyotrophic lateral sclerosis) OR TS=(Lou Gehrig* disease) OR TS=(Lou Gehrig* syndrome) OR TS=(charcot disease) 487

## National Institute of Informatics Support Academic Information Services (CiNii)

("motor neuron disease" OR "amyotrophic lateral sclerosis" OR "Lou Gehrig* disease" OR "Lou Gehrig* syndrome" OR "charcot disease") AND ("Phytotherapy" OR "Traditional medicine" OR "medicinal plant" OR "herb*" OR "plant extract" OR "plant preparation" OR "natural compound*" OR "kampo medicine" OR "Chinese materia medica" OR "Japanese medicine" OR "Japanese drug" OR "Japanese formul*" OR "Japanese prescri*") AND ("random*") 0

## SinoMed

("运动神经元病"[常用字段:智能] OR "肌萎缩侧索硬化"[常用字段:智能] OR "肌萎缩侧索硬化症"[常用字段:智能]) AND ("随机"[全部字段:智能]) AND ("草药"[常用字段:智能] OR "中医药"[常用字段:智能] OR "中药"[常用字段:智能] OR "中医"[常用字段:智能] OR "中成药"[常用字段:智能] OR "饮片"[常用字段:智能] OR "植物药"[常用字段:智能] OR "中西医结合"[常用字段:智能] OR "方"[标题:智能] OR "汤"[标题:智能] OR "散"[标题:智能] OR "丸"[标题:智能] OR "法"[标题:智能] OR "饮"[标题:智能] OR "膏"[标题:智能] OR "片"[标题:智能] OR "注射液"[标题:智能] OR "颗粒"[标题:智能]) 61

## Chinese National Knowledge Infrastructure Database (CNKI):

(SU%='运动神经元病' OR SU%='肌萎缩侧索硬化' OR SU%='肌萎缩侧索硬化症') AND (FT='随机') AND (SU%='草药' OR SU%='中医药' OR SU%='中药' OR SU%='中成药' OR TKA='中医' OR TKA='植物药' OR TKA='饮片' OR TKA='中西医结合' OR TI='方' OR TI='汤' OR TI='散' OR TI='丸' OR TI='法' OR TI='饮' OR TI='膏' OR TI='片' OR TI='注射液' OR TI='颗粒') 145

## Wanfang data

(主题:("运动神经元病") or 主题:("肌萎缩侧索硬化") or 主题:("肌萎缩侧索硬化症")) and (全部:("随机")) and (主题:("草药") or 主题:("中医药") or 主题:("中药") or 主题:("中成药") or 题名或关键词:("中医") or 题名或关键词:("植物药") or 题名或关键词:("饮片") or 题名或关键词:("中西医结合") or 题名或关键词:("方") or 题名或关键词:("汤") or 题名或关键词:("散") or 题名或关键词:("丸") or 题名或关键词:("法") or 题名或关键词:("饮") or 题名或关键词:("膏") or 题名或关键词:("片") or 题名或关键词:("注射液") or 题名或关键词:("颗粒")) 114

# Appendix 2. Characteristics of excluded studies.

| **Study** | **Reason for exclusion** |
| --- | --- |
| Bao 2014 | One of multiple publications of same study; no full-text. |
| Bisordi 2016 | Conference abstract; no relevant data. |
| Caldarazzo 2016 | Conference abstract; no relevant data. |
| Cao 2016 | Did not address outcome concerned. |
| Chen 2004 | This study included juveniles. The participants did not meet the inclusion criteria. |
| Chen 2005a | Did not address outcome concerned. |
| Chen 2005b | This study included juveniles. The participants did not meet the inclusion criteria. |
| Chen 2005c | Did not address outcome concerned. |
| Chen 2012 | Did not address outcome concerned. |
| Chico 2018b | Duplicate publication failed to recognize by Automation tools. |
| Ding 2016 | Clinical observational study. The study design did not meet the inclusion criteria. |
| Huang 2015 | Did not address outcome concerned. |
| Li 2009 | Duplicate publication failed to recognize by Automation tools. |
| Liu 2005 | Did not address outcome concerned. |
| Mu 2015 | Did not address outcome concerned. |
| Pan 2013c | Duplicate publication failed to recognize by Automation tools. |
| Pan 2013d | Duplicate publication failed to recognize by Automation tools. |
| Ren 2008 | One of publications of a study included; no full-text. |
| Shen 2012 | Did not address outcome concerned. |
| Tang 1997 | This study included juveniles. The participants did not meet the inclusion criteria. |
| Wang 2010 | Conference abstract; no relevant data. |
| Wang 2014 | Did not address outcome concerned. |
| Wu 2016a | Did not address outcome concerned. |
| Wu 2016b | Did not address outcome concerned. |
| Yang 2015 | Did not address outcome concerned. |
| Yuan 2005 | Did not address outcome concerned. |
| Zhang 2015 | Did not address outcome concerned. |
| Zhu 2010 | Conference abstract; no relevant data. |
| Zhu 2012 | Conference abstract; no relevant data. |
| Zhu 2017a | The lower limit of age is unclear. The participants did not meet the inclusion criteria. |
| Zhu 2017b | Duplicate publication failed to recognize by Automation tools. |

# Appendix 3. The chemical and botanical characterisation of the involved preparation according to the “Four Pillars of Best Practice”.

(A)
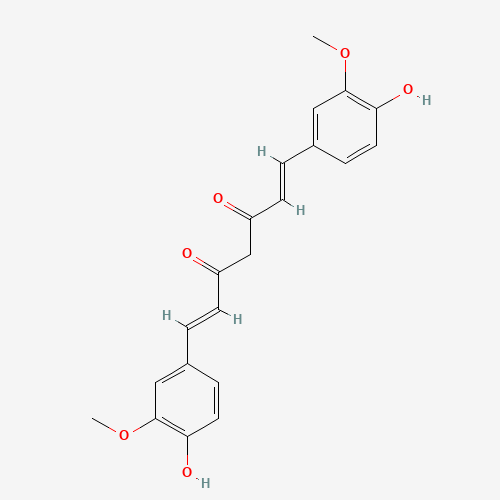
 (B)
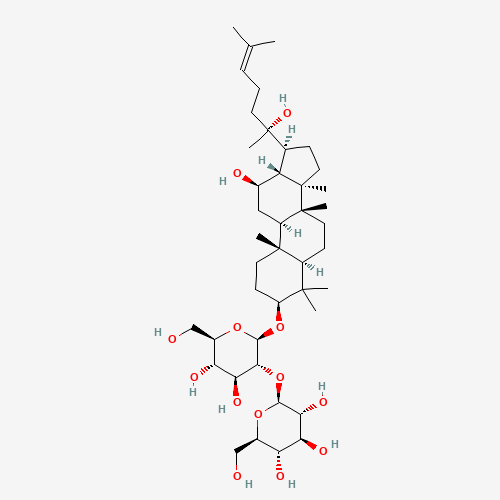
 (C)
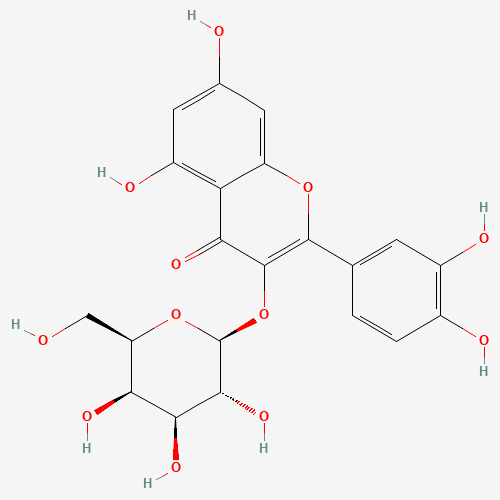
 (D)


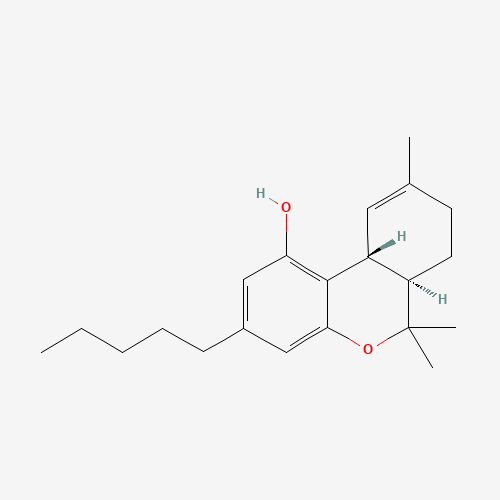


(E)
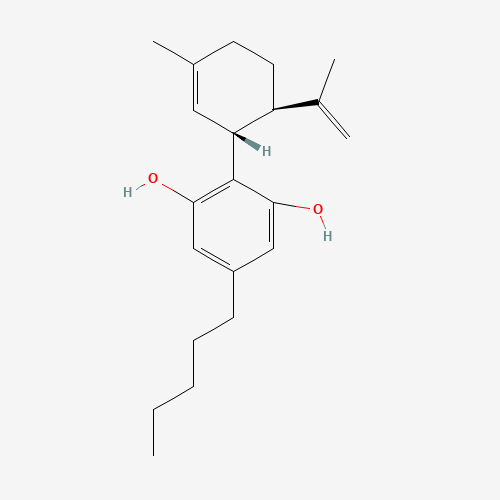
 (F)
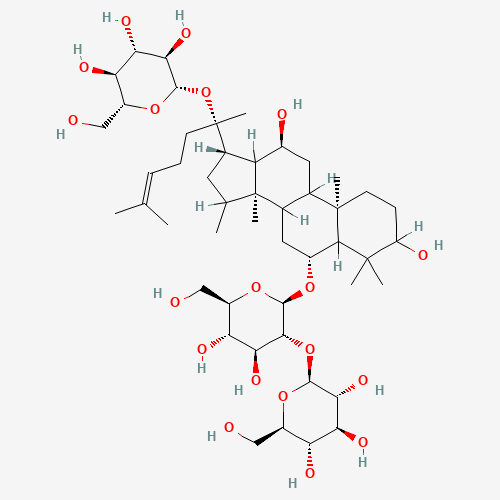
 (G)
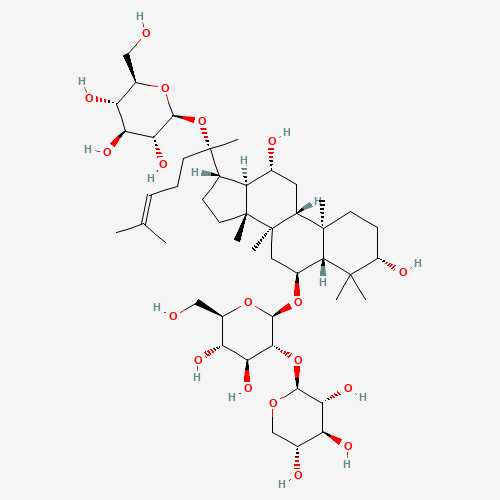
 (H)
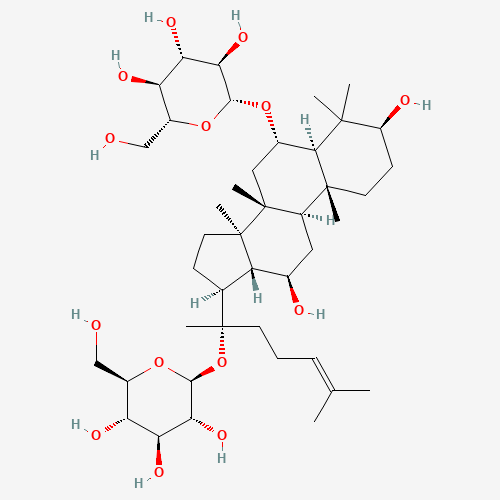


(I)
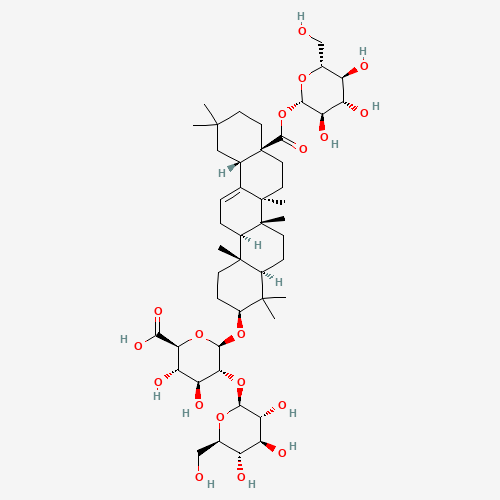
 (J)
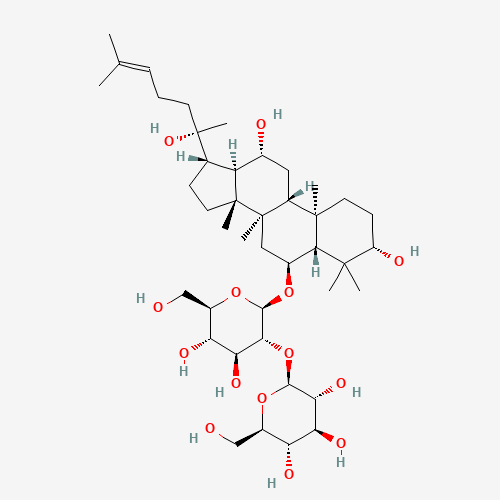
 (K)
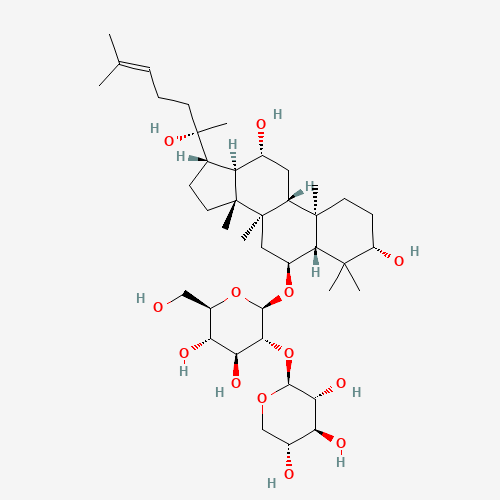
 (L)
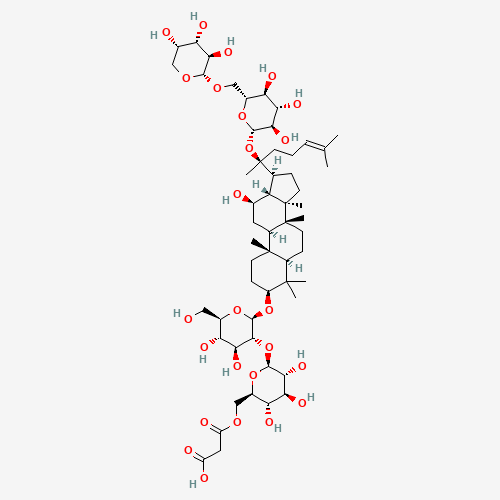


(M)
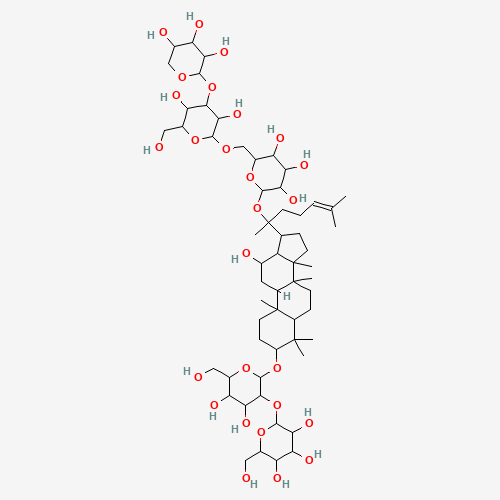
 (N)
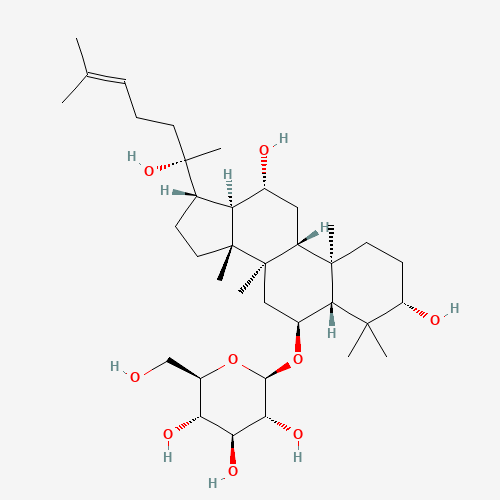
 (O)
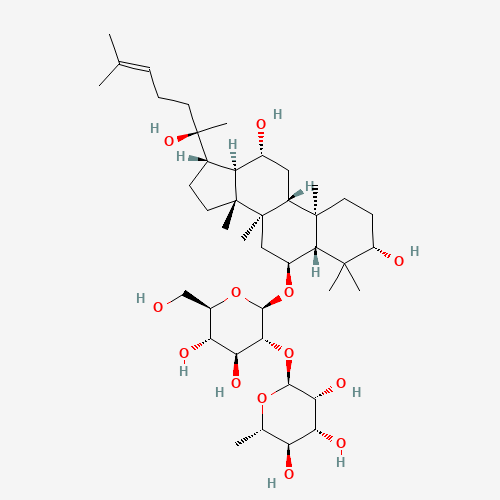
 (P)
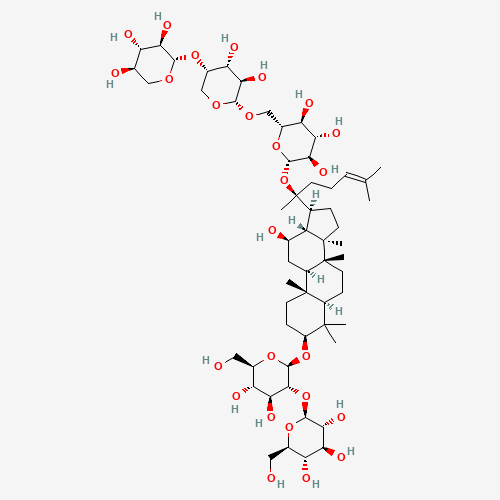
 (Q)
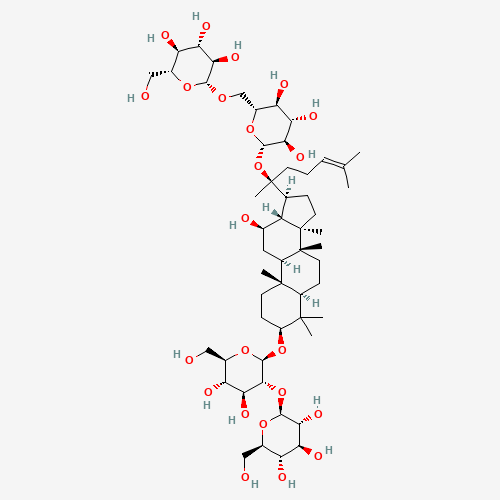
 (R)
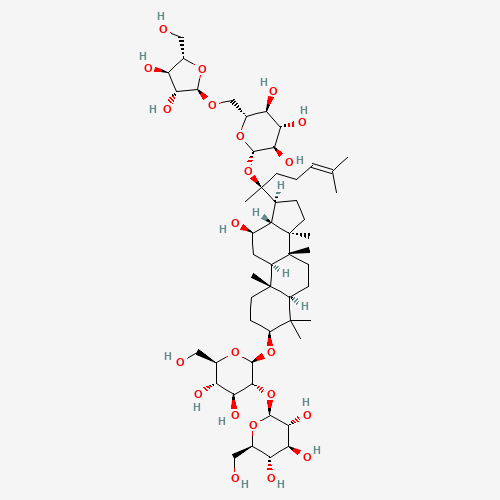
 (S)
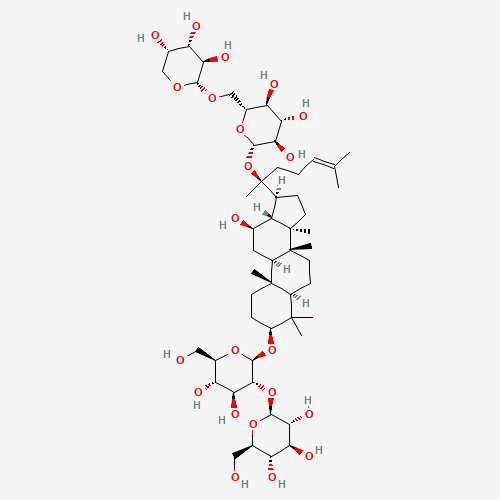
 (T)
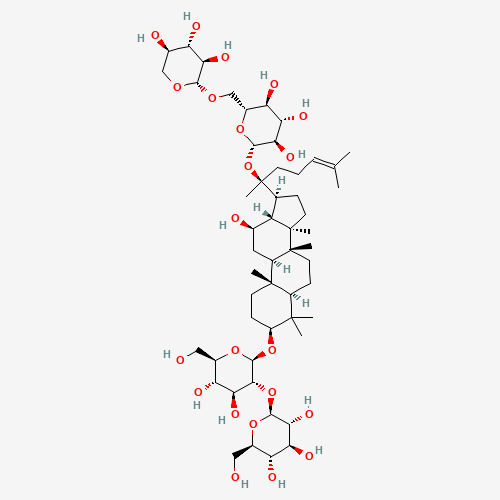


(U)
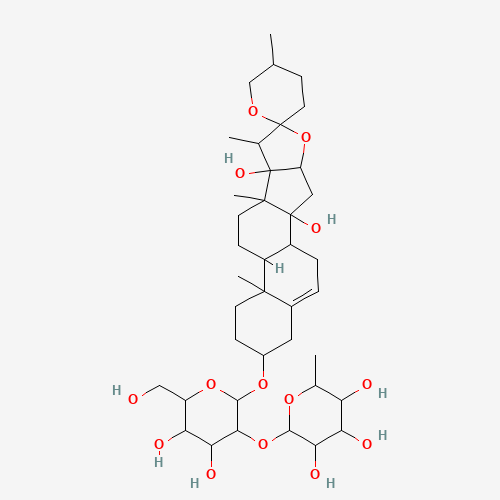
 (V)
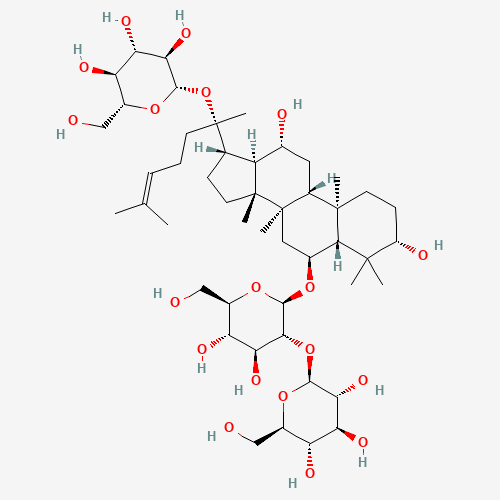
 (W)
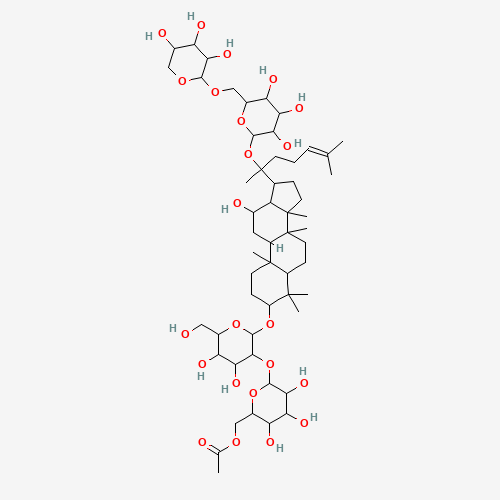
 (X)
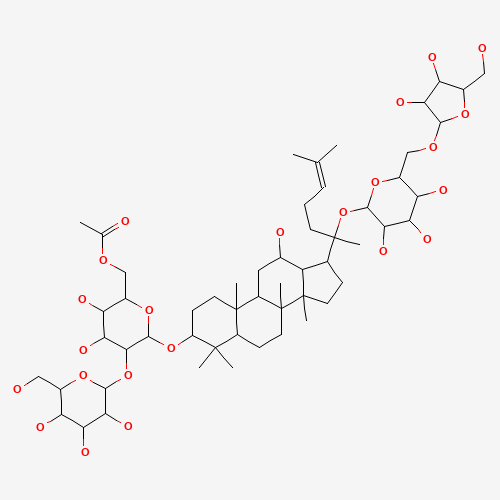


(Y)
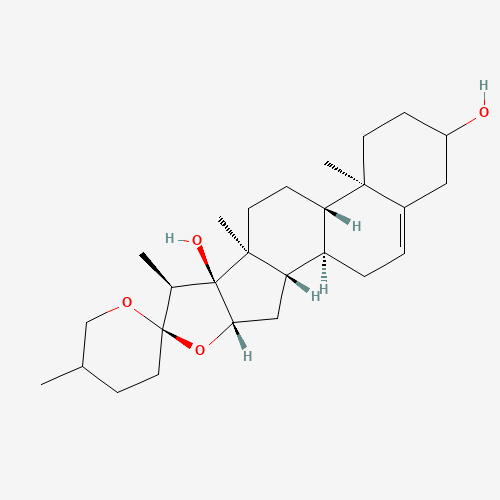
 (Z)
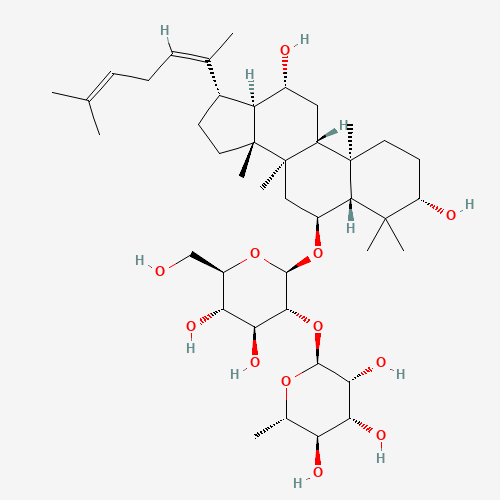
 (AA)
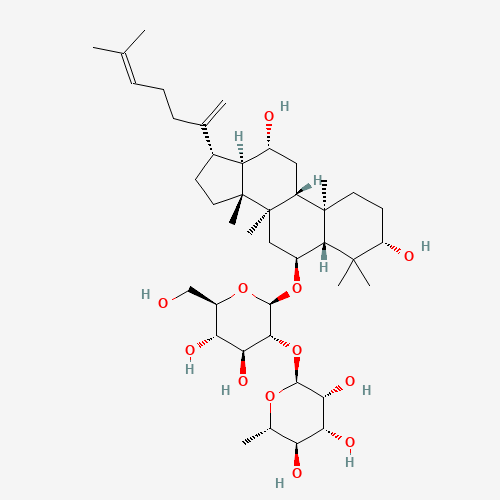
 (AB)
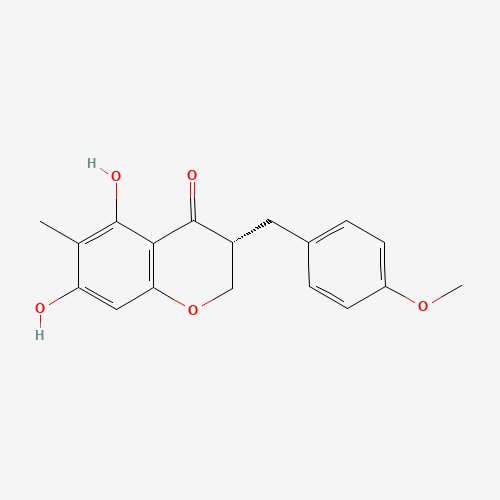


(AC)
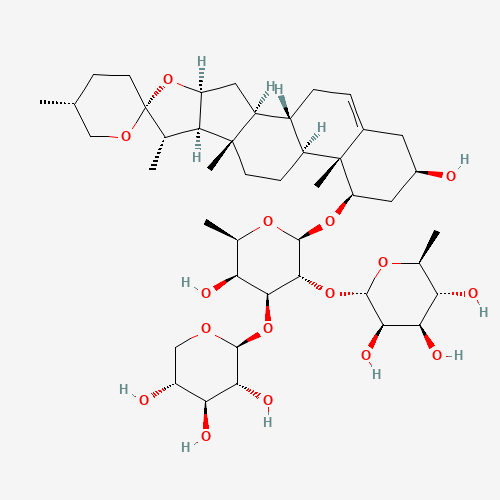
 (AD)
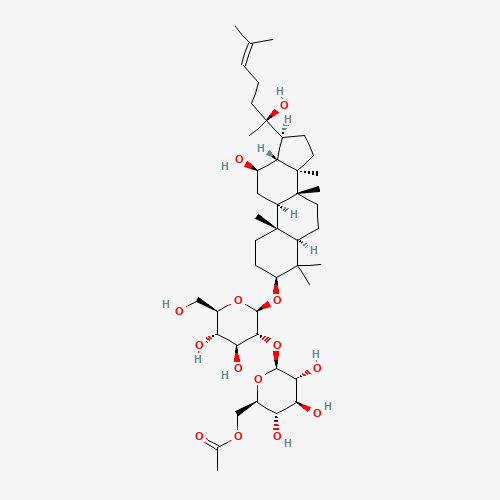
 (AE)
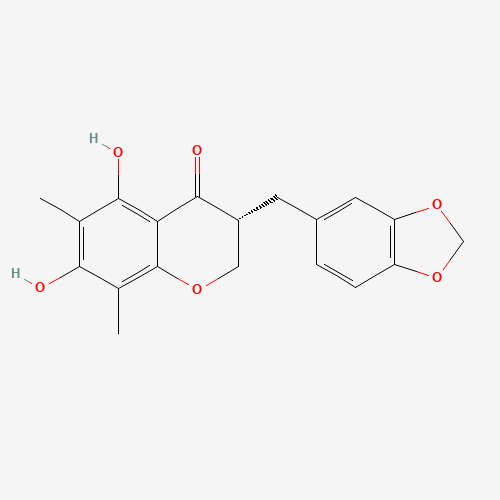
 (AF)
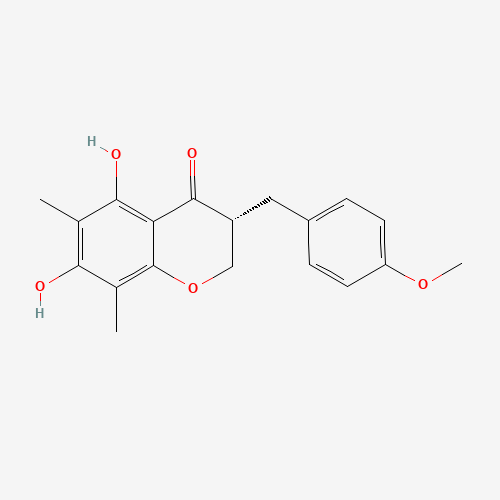


(AG)
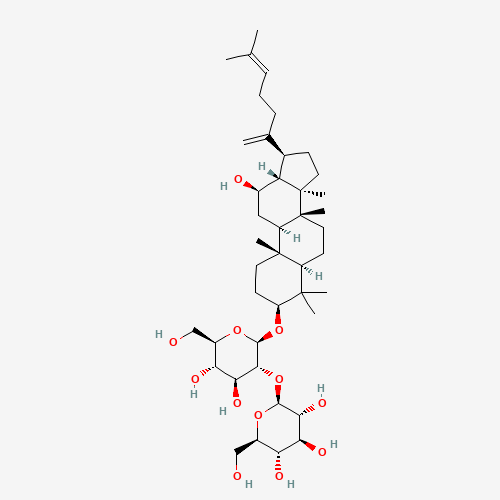
 (AH)
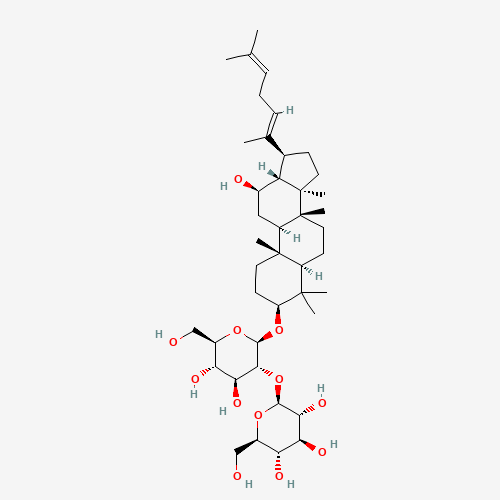
 (AI)
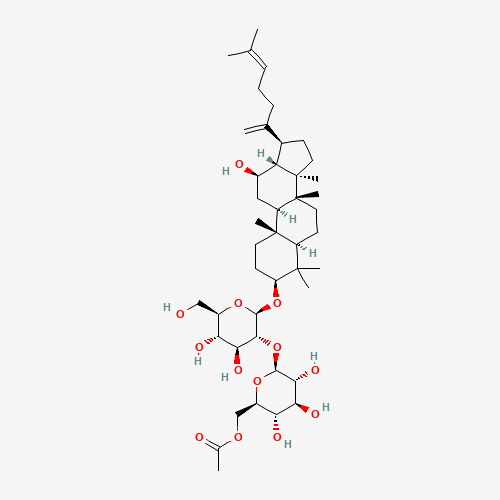


## Figure 1. The structure of active compounds included in this systematic review. (A) Curcumin. (B) (20R)-Ginsenoside Rg3 in *Jiweiling* injection. (C) Hyperoside in *Jiweiling* injection. (D) Delta­9­tetrahydrocannabinol in Nabiximols. (E) Cannabidiol in Nabiximols. (F) 20-glucoginsenoside Rf in *Shenmai* injection. (G) Notoginsenoside R1 in *Shenmai* injection. (H) Ginsenoside Rg1 in *Shenmai* injection. (I) Ginsenoside Ro in *Shenmai* injection. (J) Ginsenoside Rf in *Shenmai* injection. (K) Notoginsenoside R2 in *Shenmai* injection. (L) Malonyl-ginsenoside Rb2 in *Shenmai* injection. (M) Ginsenoside Ra3 in *Shenmai* injection. (N) Ginsenoside Rh1 in *Shenmai* injection. (O) 20R-Ginsenoside Rg2 in *Shenmai* injection. (P) Ginsenoside Ra1 in *Shenmai* injection. (Q) Ginsenoside Rb1 in *Shenmai* injection. (R) Ginsenoside Rc in *Shenmai* injection. (S) Ginsenoside Rb2 in *Shenmai* injection. (T) Ginsenoside Rb3 in *Shenmai* injection. (U) Ophiogenin-3-O-alpha-L-rhaMnopyranosyl-(1-->2)-beta-D-glucopyranoside in *Shenmai* injection. (V) ginsenoside-Rd in *Shenmai* injection. (W) Ginsenoside Rs1 in *Shenmai* injection. (X) Ginsenoside Rs2 in *Shenmai* injection. (Y) Spirost-5-ene-3,17-diol in *Shenmai* injection. (Z) Ginsenoside Rg4 in *Shenmai* injection. (AA) Ginsenoside Rg3 in *Shenmai* injection. (AB) Ophiopogonanone B in *Shenmai* injection. (AC) Ophiopogonin D in *Shenmai* injection. (AD) Ginsenoside Rs3 in *Shenmai* injection. (AE) Methylophiopogonanone A in *Shenmai* injection. (AF) Methylophiopogonanone B in *Shenmai* injection. (AG) Ginsenoside Rk1 in *Shenmai* injection. (AH) Ginsenoside Rg5 in *Shenmai* injection. (AI) Ginsenoside Rs5 in *Shenmai* injection.

## Table 1. Multiherbal

| **Study** | **Species, source, concentration** | **Quality control reported? (Y/N)** | **Chemical analysis reported? (Y/N)** |
| --- | --- | --- | --- |
| Bao 2016 | - Root of Codonopsis pilosula (Franch.) Nannf., 20g - Root of Astragalus mongholicus Bunge, 20g - Rhizome of Atractylodes macrocephala Koidz., 10g - Fruit of Poria cocos (Schw.) Wolf., 10g - Root of Glycyrrhiza glabra L., 10g - Root of Angelica sinensis (Oliv.) Diels, 10g | Y-Prepared according to Chinese pharmacopeia | Y- UPLC-PDA |
| Fang 2016 | - Root of Astragalus mongholicus Bunge, [Jiangyin Tianjiang Pharmaceutical Co., Ltd] - Root of Codonopsis pilosula (Franch.) Nannf., [Jiangyin Tianjiang Pharmaceutical Co., Ltd] - Rhizome of Atractylodes macrocephala Koidz., [Jiangyin Tianjiang Pharmaceutical Co., Ltd] - Fruit of Poria cocos (Schw.) Wolf., [Jiangyin Tianjiang Pharmaceutical Co., Ltd] - Root tuber of Ophiopogon japonicus (Thunb.) Ker Gawl., [Jiangyin Tianjiang Pharmaceutical Co., Ltd] - Fruit of Schisandra chinensis (Turcz.) Baill., [Jiangyin Tianjiang Pharmaceutical Co., Ltd] - Peel of Citrus deliciosa Ten., [Jiangyin Tianjiang Pharmaceutical Co., Ltd] - Tuber of Pinellia ternata (Thunb.) Makino, [Jiangyin Tianjiang Pharmaceutical Co., Ltd] - Rhizome of Iris domestica (L.) Goldblatt & Mabb., [Jiangyin Tianjiang Pharmaceutical Co., Ltd] - Rhizome of Acorus calamus var. angustatus Besser, [Jiangyin Tianjiang Pharmaceutical Co., Ltd] - Batryticated silkworm, [Jiangyin Tianjiang Pharmaceutical Co., Ltd] - Tuber of Sauromatum giganteum (Engl.) Cusimano & Hett., [Jiangyin Tianjiang Pharmaceutical Co., Ltd] - Seed of Strychnos nux-vomica L., [Jiangyin Tianjiang Pharmaceutical Co., Ltd] - Root of Glycyrrhiza glabra L., [Jiangyin Tianjiang Pharmaceutical Co., Ltd] - Drug Specifications: 50mg per bag | Y-Prepared according to Chinese pharmacopeia | Y- HPLC-ELSD |
| Jin 2013 | - Cornu Cervi Degelatinatum, 12g - Phyllostachys edulis (Carrière) J.Houz., 20g - Root of Panax ginseng C.A.Mey., 15g - Barbary boxthorn of Lycium barbarum L., 20g | Y-Prepared according to Chinese pharmacopeia | Y-HPLC |
| Li 2011 | - Root of Astragalus mongholicus Bunge - Rhizome of Atractylodes macrocephala Koidz. - Fruit of Cornus officinalis Siebold & Zucc. | Y-Prepared according to Chinese pharmacopeia | Y-HPLC |
| Li 2019 | - Root of Astragalus mongholicus Bunge, 30-60g - Root of Codonopsis pilosula (Franch.) Nannf., 15-30g - Rhizome of Atractylodes macrocephala Koidz., 15g - Bark of Eucommia ulmoides Oliv., 12g - Stem of Cassytha filiformis L., 12g - Root tuber of Ophiopogon japonicus (Thunb.) Ker Gawl., 9g - Tuber of Pinellia ternata (Thunb.) Makino, 9g - Root of Aster tataricus L.f., 9g - Bitter apricot seed of apricot kernel, 9g - Root of Platycodon grandiflorus (Jacq.) A.DC., 9g - Dried rhizome of Zingiber officinale Roscoe, 6g - Fruit of Schisandra chinensis (Turcz.) Baill., 6g - Peel of Citrus deliciosa Ten., 6g - Batryticated silkworm, 6g - Root of Bupleurum falcatum L., 6g - Root of Glycyrrhiza glabra L., 6g - Seed of Strychnos nux-vomica L., 0.1g | Y-Prepared according to Chinese pharmacopeia | Y- HPLC-ELSD |
| Pan 2013 | - Root fo Panax ginseng C.A.Mey., 9g - Root of Astragalus mongholicus Bunge, 30g - Stem of Cistanche deserticola Ma, 12g - Rhizome of Atractylodes macrocephala Koidz., 9g - Fruit of Poria cocos (Schw.) Wolf., 9g - Root of Glycyrrhiza glabra L., 9g | Y-Prepared according to Chinese pharmacopeia | Y- UPLC-PDA |
| Pan 2015 | - Manufactured by Shijiazhuang Yiling Pharmaceutical Co., Ltd - Drug Specifications: 50mg per bag | Y-Prepared according to Chinese pharmacopeia | Y-HPLC |
| Su 2006 | - Root of Astragalus mongholicus Bunge, 60g - Root of Codonopsis pilosula (Franch.) Nannf., 30g - Aerial parts of Epimedium sagittatum (Siebold & Zucc.) Maxim., 30g - Fruit of Poria cocos (Schw.) Wolf., 20g - Chinese foxglove root of Poria cocos Rehmannia glutinosa (Gaertn.) DC., 20g - Root of Rheum officinale Baill., 6g - Root of Actaea racemosa L., 20g - Root of Glycyrrhiza glabra L., 10g | Y-Prepared according to Chinese pharmacopeia | Y-HPLC |
| Sui 2016 | - Aerial parts of Epimedium sagittatum (Siebold & Zucc.) Maxim. - Root of Astragalus mongholicus Bunge - Rhizome of Atractylodes macrocephala Koidz. - Fruit of Cornus officinalis Siebold & Zucc. - Chinese foxglove root of Poria cocos Rehmannia glutinosa (Gaertn.) DC. - Drug Specifications: 10g per bag | Y-Prepared according to Chinese pharmacopeia | Y-HPLC, TLC |
| Wang 2009 | - Root of Astragalus mongholicus Bunge - Rhizome of Atractylodes macrocephala Koidz. - Fruit of Cornus officinalis Siebold & Zucc. | Y-Prepared according to Chinese pharmacopeia | Y-HPLC |
| Wang 2017a | - Root of Astragalus mongholicus Bunge, [Jiangyin Tianjiang Pharmaceutical Co., Ltd] 30g - Root of Codonopsis pilosula (Franch.) Nannf., [Jiangyin Tianjiang Pharmaceutical Co., Ltd] 15g - Rhizome of Atractylodes macrocephala Koidz., [Jiangyin Tianjiang Pharmaceutical Co., Ltd] 15g - Fruit of Schisandra chinensis (Turcz.) Baill., [Jiangyin Tianjiang Pharmaceutical Co., Ltd] 10g - Bark of Eucommia ulmoides Oliv., [Jiangyin Tianjiang Pharmaceutical Co., Ltd] 10g - Stem of Cassytha filiformis L., [Jiangyin Tianjiang Pharmaceutical Co., Ltd] 10g - Root tuber of Ophiopogon japonicus (Thunb.) Ker Gawl., [Jiangyin Tianjiang Pharmaceutical Co., Ltd] 10g - Peel of Citrus deliciosa Ten., [Jiangyin Tianjiang Pharmaceutical Co., Ltd] 10g - Tuber of Pinellia ternata (Thunb.) Makino, [Jiangyin Tianjiang Pharmaceutical Co., Ltd] 10g - Batryticated silkworm, [Jiangyin Tianjiang Pharmaceutical Co., Ltd] 10g - bitter apricot seed of apricot kernel, [Jiangyin Tianjiang Pharmaceutical Co., Ltd] 10g - Root of Platycodon grandiflorus (Jacq.) A.DC., [Jiangyin Tianjiang Pharmaceutical Co., Ltd] 5g - Root of Bupleurum falcatum L., [Jiangyin Tianjiang Pharmaceutical Co., Ltd] 5g - Seed of Strychnos nux-vomica L., [Jiangyin Tianjiang Pharmaceutical Co., Ltd] 0.2g - Root of Glycyrrhiza glabra L., [Jiangyin Tianjiang Pharmaceutical Co., Ltd] 10g | Y-Prepared according to Chinese pharmacopeia | Y- HPLC-ELSD |
| Wang 2017b | - Chinese foxglove root of Poria cocos Rehmannia glutinosa (Gaertn.) DC., 30g - Root of Gynochthodes officinalis (F.C.How) Razafim. & B.Bremer, 15g - Stem of Dendrobium nobile Lindl., 20g - Stem of Cistanche deserticola Ma, 30g - Fruit of Schisandra chinensis (Turcz.) Baill., 20g - Root tuber of Ophiopogon japonicus (Thunb.) Ker Gawl., 20g - Root of Astragalus mongholicus Bunge, 30g - Rhizome of Atractylodes macrocephala Koidz., 30g - Peel of Citrus deliciosa Ten., 15g - Root of Bupleurum falcatum L., 10g - Rhizome of Acorus calamus var. angustatus Besser, 12g - Root of Polygala tenuifolia Willd., 10g - Aerial parts of Mentha canadensis L., 10g - Root of Platycodon grandiflorus (Jacq.) A.DC., 20g - Fresh tubers of Zingiber officinale Roscoe, 3 pieces | Y-Prepared according to Chinese pharmacopeia | Y-HPLC |
| Xv 2011 | - Root of Astragalus mongholicus Bung - Chinese foxglove root of Poria cocos Rehmannia glutinosa (Gaertn.) DC. - Aerial parts of Epimedium sagittatum (Siebold & Zucc.) Maxim. - Root of Gynochthodes officinalis (F.C.How) Razafim. & B.Bremer - Fruit of Cornus officinalis Siebold & Zucc. - Fruit of Poria cocos (Schw.) Wolf. - Stem of Dendrobium nobile Lindl. - Root of Achyranthes bidentata Blume - Root of Bupleurum falcatum L. - Rhizome of Curcuma longa L. - Drug Specifications: 50mg per bag | Y-Prepared according to Chinese pharmacopeia | Y-HPLC |
| Zhu 2016 | - Root of Codonopsis pilosula (Franch.) Nannf., 20g - Root of Astragalus mongholicus Bunge, 20g - Rhizome of Atractylodes macrocephala Koidz., 10g - Fruit of Poria cocos (Schw.) Wolf., 10g - Root of Glycyrrhiza glabra L., 10g - Root of Angelica sinensis (Oliv.) Diels, 10g | Y-Prepared according to Chinese pharmacopeia | Y- UPLC-PDA |

## Table 2. Patented formulations, botanical or chemical

| **Study** | **Formulation** | **Source** | **Species, concentration** | **Quality control reported? (Y/N)** | **Chemical anaysis reported? (Y/N)** |
| --- | --- | --- | --- | --- | --- |
| Cai 2011 | *Shenmai* injection | Chiatai Qingchunbao Pharmaceutical Co., Ltd. | - Root of Panax ginseng C.A.Mey. - Root tuber of Ophiopogon japonicus (Thunb.) Ker Gawl. - Drug Specifications: 5ml per vial | Y-Prepared according to Chinese pharmacopeia | Y-HPLC |
| Chico 2018 | Curcumin | Alveda Laboratories s.r.l | - Diferuloylmethane, 600mg | Y-Prepared according to patent (Italy) | Y-HPLC |
| Ma 2006 | *Jiweiling* injection | Shijiazhuang Yiling Pharmaceutical Co., Ltd | - Root fo Panax ginseng C.A.Mey. - Root of Angelica sinensis (Oliv.) Diels - Drug Specifications: 8ml per ampoule; including 0.9g rude medicine per 1mL | Y-Prepared according to Chinese pharmacopeia | Y-HPLC-MS |
| Riva 2019 | Nabiximols | GW Pharmaceuticals Ltd. | - Delta­-9-­tetrahydrocannabinol, 2.7mg per 100 µL - Cannabidiol, 2.5mg per 100 µL | Y-Prepared according to European pharmacopeia | Y-HPLC |
| Wang 2007 | *Jiweiling* injection | Shijiazhuang Yiling Pharmaceutical Co., Ltd | - Root fo Panax ginseng C.A.Mey. - Root of Angelica sinensis (Oliv.) Diels - Drug Specifications: 8ml per ampoule; including 0.9g rude medicine per 1mL | Y-Prepared according to Chinese pharmacopeia | Y-HPLC-MS |
| Zhang 2020 | *Shenmai* injection | Chiatai Qingchunbao Pharmaceutical Co., Ltd. | - Root of Panax ginseng C.A.Mey. - Root tuber of Ophiopogon japonicus (Thunb.) Ker Gawl. - Drug Specifications: 5ml per vial | Y-Prepared according to Chinese pharmacopeia | Y-HPLC |

# Appendix 4. Outcome data

## Activity limitation data

| **Study** | **Treatment Mean** | **Treatment SD** | **Control Mean** | **Control SD** | **Treatment N** | **Control N** | **Treatment** | **Control** | **Outcome measures** |
| --- | --- | --- | --- | --- | --- | --- | --- | --- | --- |
| Cai 2011 | 31.12 | 4.11 | 27.02 | 3.23 | 20 | 18 | *Shenmai* injection + conventional treatment | conventional treatment | ALSFRS/ALSFRS-R^a^ |
| Jin 2013 | 5.27 ^b^ | 2.09 ^b^ | 7.08 ^b^ | 2.43 ^b^ | 15 | 13 | *Guilu Erxian* glues + acupuncture + Riluzole | Riluzole | ALSFRS-R |
| Ma 2006 | 25.62 | 5.18 | 22.84 | 5.22 | 30 | 30 | *Jiweiling* injection + dummy Riluzole | Riluzole + dummy *Jiweiling* injection | ALSFRS |
|  | 76.61 | 12.1 | 69.42 | 19.38 |  |  |  |  | modified Norris scale |
| Pan 2015 | 3.12 | 0.65 | 2.44 | 0.57 | 38 | 38 | *Shenzhe Jiangqi* powder + dummy Riluzole | Riluzole + dummy *Shenzhe Jiangqi* powder | ALSFRS |
|  | 3.51 | 0.61 | 2.92 | 0.58 |  |  |  |  | modified Norris scale |
| Zhang 2020 | 31.26 | 4.12 | 27.24 | 3.31 | 42 | 42 | *Shenmai* injection + conventional treatment | conventional treatment | ALSFRS-R |
| Zhu 2016 | 33 | 8.05 | 28 | 9.18 | 24 | 21 | *Jiawei Sijunzi* decoction | 1/10 dose of Jiawei Sijunzi decoction | ALSFRS-R |
| Wang 2017a | 35.9 | 5.5 | 32.4 | 5.7 | 28 | 26 | *Jianpi Yifei* decoction + Riluzole | Riluzole | ALSFRS-R |
| Wang 2007 | 25.37 | 4.28 | 22.5 | 3.86 | 30 | 30 | *Jiweiling* injection | Riluzole | ALSFRS |
| Li 2011 | -9.37 ^b^ | 2.37 ^b^ | -11.29 ^b^ | 3.7 ^b^ | 30 | 28 | *Fuyuan Shengji* granule + Riluzole | Riluzole | modified Norris scale |
| Chico 2018 | NA | NA | NA | NA | NA | NA | Curcumin | placebo | ALSFRS-R |
| Fang 2016 | 36.4 | 7 | 33.9 | 6.9 | 18 | 19 | *Jianpi Yifei* decoction | Riluzole | ALSFRS-R |
| Pan 2013 | 34.4 | 7.9 | 30.6 | 9.1 | 23 | 19 | *Jiawei Sijunzi* decoction | Riluzole | ALSFRS-R |
| Riva 2019 | -0.1 ^b^ | 1.32 ^b^ | -0.7 ^b^ | 1.82 ^b^ | 29 | 30 | Cannabinoids | placebo | ALSFRS-R |
| Li 2019 | 25.81 | 6.41 | 22.46 | 6.12 | 39 | 39 | *Jianpi Yifei* decoction + massage + conventional treatment | conventional treatment | ALSFRS |
| Wang 2017b | 29.16 | 6.22 | 29.73 | 6.54 | 30 | 30 | *Zishen Jianpi* decoction + conventional treatment | conventional treatment | ALSFRS |
| Su 2006 | 1 ^b^ | 6.4 ^b^ | -1.9 ^b^ | 4.1 ^b^ | 25 | 10 | *Yiqi Qiangji* decoction + Riluzole | Riluzole | modified Norris scale |
| Sui 2016 | 68.82 | 15.9 | 72.16 | 15.36 | 33 | 31 | *Huoling Shengji* decoction | Riluzole | modified Norris scale |
| Wang 2009 | 71.8 | 22.51 | 71.08 | 18.6 | 100 | 25 | *Fuyuan Shengji* granule | Riluzole | modified Norris scale |
| Xv 2011 | 82^c^ | 22.75 ^c^ | 75.5 ^c^ | 27 ^c^ | 40 | 40 | *Bushen Jianpi Shugan* decoction | Riluzole | modified Norris scale |

*ALSFRS, Amyotrophic Lateral Sclerosis Functional Rating Scale; ALSFRS-R, Amyotrophic Lateral Sclerosis Functional Rating Scale-Revised.*

*^a^ The version of ALS functional rating scale is unclear; ^b^ reported in mean change and standard deviation for change from baseline; ^c^ reported in median and interquartile range.*

## Survival data

| **Study** | **Treatment survival rate** | **Treatment N** | **Control survival rate** | **Control N** | **Follow-up** | **Hazard ratio** | **Significant** | **95% CI** | **Treatment** | **Control** |
| --- | --- | --- | --- | --- | --- | --- | --- | --- | --- | --- |
| Li 2011 | 80% | 30 | 71. 4% | 28 | 18 months | Unclear | Unclear | Unclear | *Fuyuan Shengji* granule + Riluzole | Riluzole |

*CI, confidence interval.*

## Loss of strength data

| **Study** | **Treatment Mean** | **Treatment SD** | **Control Mean** | **Control SD** | **Treatment N** | **Control N** | **Treatment** | **Control** | **Outcome measures** |
| --- | --- | --- | --- | --- | --- | --- | --- | --- | --- |
| Ma 2006 | 73.74 | 9.46 | 66.48 | 7.62 | 30 | 30 | *Jiweiling* injection + dummy Riluzole | Riluzole + dummy *Jiweiling* injection | FVC |
|  | 75.64 | 7.55 | 67.12 | 8.38 | 30 | 30 |  |  | VC |
| Wang 2007 | 67.54 | 12.49 | 68.17 | 12.68 | 30 | 30 | *Jiweiling* injection | Riluzole | FVC |
|  | 70.42 | 10.27 | 69.04 | 7.34 | 30 | 30 |  |  | VC |
| Li 2011 | 13.34 ^a^ | 2.44 ^a^ | 12.5 ^a^ | 2.36 ^a^ | 30 | 28 | *Fuyuan Shengji* granule + Riluzole | Riluzole | FVC |
| Pan 2013 | 3.5 | 0.7 | 3.5 | 0.4 | 23 | 19 | *Jiawei Sijunzi* decoction | Riluzole | MRC |
| Wang 2017a | 77.35 | 19.38 | 75.21 | 18.36 | 28 | 26 | *Jianpi Yifei* decoction + Riluzole | Riluzole | FVC |
|  | 8.46 | 2.01 | 8.01 | 2.12 | 28 | 26 |  |  | MRC |
| Chico 2018 | NA | NA | NA | NA | 15 | 21 | Curcumin | placebo | HHD |
|  | NA | NA | NA | NA | 15 | 21 |  |  | MRC |
| Riva 2019 | 0.57 ^a^ | 9.21 ^a^ | -6.85 ^a^ | 11 ^a^ | 29 | 30 | Cannabinoids | placebo | FVC |
|  | -0.79 ^a^ | 8.83 ^a^ | 0.03 ^a^ | 1.13 ^a^ | 29 | 30 |  |  | MRC |

*FVC, forced vital capacity; VC, vital capacity; MRC, Medical Research Council Scale; HHD, hand-held dynamometry.*

*^a^ Reported in mean change and standard deviation for change from baseline.*

## Quality of life data

| **Study** | **Treatment Mean** | **Treatment SD** | **Control Mean** | **Control SD** | **Treatment N** | **Control N** | **Treatment** | **Control** | **Outcome measures** |
| --- | --- | --- | --- | --- | --- | --- | --- | --- | --- |
| Pan 2015 | 7.39 | 1.31 | 6.97 | 1.26 | 38 | 38 | *Shenzhe Jiangqi* powder + dummy Riluzole | Riluzole + dummy *Shenzhe Jiangqi* powder | ALSAQ-40 |
| Ma 2006 | 104.16 | 22.47 | 90.84 | 29.04 | 30 | 30 | *Jiweiling* injection + dummy Riluzole | Riluzole + dummy *Jiweiling* injection | ALSAQ-40 |
| Wang 2007 | 96.33 | 10.86 | 93 | 17.44 | 30 | 30 | *Jiweiling* injection | Riluzole | ALSAQ-40 |
| Wang 2017b | 89.83 | 7.59 | 86.16 | 6.65 | 30 | 30 | *Zishen Jianpi* decoction + conventional treatment | conventional treatment | Barthel index |
| Fang 2016 | 38.3 | 12.5 | 36.7 | 11 | 18 | 19 | *Jianpi Yifei* decoction | Riluzole | ALSAQ-40 (sub) |
| Riva 2019 | -0.34 ^a^ | 2.97 ^a^ | -1 ^a^ | 7.47 ^a^ | 29 | 30 | Cannabinoids | placebo | Barthel index |
| Pan 2013 | 38.9 | 4.9 | 37.6 | 7.7 | 23 | 19 | *Jiawei Sijunzi* decoction | Riluzole | SF-36 (sub) |

*ALSAQ-40, Amyotrophic Lateral Sclerosis Assessment Questionnaire-40; SF-36, MOS Item Short-Form Health Survey-36.*

*^a^ Reported in mean change and standard deviation for change from baseline.*

## Functional status data

| **Study** | **Treatment Mean** | **Treatment SD** | **Control Mean** | **Control SD** | **Treatment N** | **Control N** | **Treatment** | **Control** | **Outcome measures** |
| --- | --- | --- | --- | --- | --- | --- | --- | --- | --- |
| Ma 2006 | 72.16 | 14.08 | 81.69 | 22.94 | 30 | 30 | *Jiweiling* injection + dummy Riluzole | Riluzole + dummy *Jiweiling* injection | AALSS |
| Li 2019 | 81.65 | 8.85 | 86.34 | 10.16 | 39 | 39 | *Jianpi Yifei* decoction + massage + conventional treatment | conventional treatment | AALSS |

*AALSS, Appel Amyotrophic Lateral Sclerosis Score.*

## Traditional medicine syndrome data

| **Study** | **Treatment Mean** | **Treatment SD** | **Control Mean** | **Control SD** | **Treatment N** | **Control N** | **Treatment** | **Control** | **Outcome measures** |
| --- | --- | --- | --- | --- | --- | --- | --- | --- | --- |
| Bao 2016 | Unclear | Unclear | Unclear | Unclear | 24 | 21 | *Jiawei Sijunzi* decoction + conventional treatment | conventional treatment | STMS |
| Jin 2013 | 5.53 ^a^ | 3.14 ^a^ | 1.08 ^a^ | 3.04 ^a^ | 15 | 13 | *Guilu Erxian* glues + acupuncture + Riluzole | Riluzole | STMS |
| Su 2006 | 4.7 ^a^ | 4.9 ^a^ | 0.8 ^a^ | 1.5 ^a^ | 25 | 10 | *Yiqi Qiangji* decoction + Riluzole | Riluzole | STMS |
| Sui 2016 | 11.48 | 6.9 | 13.94 | 5.91 | 33 | 31 | *Huoling Shengji* decoction | Riluzole | STMS |
| Wang 2009 | 11.24 | 6.88 | 15.2 | 6.55 | 100 | 25 | *Fuyuan Shengji* granule | Riluzole | STMS |
| Wang 2017b | 14.43 | 4.99 | 16.83 | 5.45 | 30 | 30 | *Zishen Jianpi* decoction + conventional treatment | conventional treatment | STMS |
| Xv 2011 | 11 ^b^ | 8.75 ^b^ | 10 ^b^ | 8 ^b^ | 40 | 40 | *Bushen Jianpi Shugan* decoction | Riluzole | STMS |
| Zhu 2016 | 8 | 3.73 | 12.09 | 5.21 | 24 | 21 | *Jiawei Sijunzi* decoction | 1/10 dose of *Jiawei Sijunzi* decoction | STMS |
| Li 2019 | 11.08 | 2.69 | 18.37 | 4.12 | 39 | 39 | *Jianpi Yifei* decoction + massage + conventional treatment | conventional treatment | STMS |

*STMS, score of traditional medicine syndrome.*

*^a^ Reported in mean change and standard deviation for change from baseline; ^b^ reported in median and interquartile range.*

## Motor neuron loss data

| **Study** | **Treatment Mean** | **Treatment SD** | **Control Mean** | **Control SD** | **Treatment N** | **Control N** | **Treatment** | **Control** | **Outcome measures** |
| --- | --- | --- | --- | --- | --- | --- | --- | --- | --- |
| Ma 2006 | 118.96 | 45.98 | 82.47 | 48.04 | 22 | 12 | *Jiweiling* injection + dummy Riluzole | Riluzole + dummy *Jiweiling* injection | MUNE |
| Wang 2007 | 176.35 | 74.96 | 148.75 | 68.01 | 30 | 30 | *Jiweiling* injection | Riluzole | MUNE (right APB) |
|  | 173 | 59.13 | 140.05 | 52.82 |  |  |  |  | MUNE (left APB) |
| Pan 2015 | 118.96 | 45.98 | 82.47 | 48.04 | 38 | 38 | *Shenzhe Jiangqi* powder + dummy Riluzole | Riluzole + dummy *Shenzhe Jiangqi* powder | MUNE |
| Wang 2009 | 54.57 | 5.06 | 53.57 | 3.96 | Unclear | Unclear | *Fuyuan Shengji* granule | Riluzole | CMAP amplitude |
| Su 2006 | 0.5 ^a^ | 1.1 ^a^ | 0.3 ^a^ | 0.4 ^a^ | 25 | 10 | *Yiqi Qiangji* decoction + Riluzole | Riluzole | CMAP amplitude |
| Li 2019 | Unclear | Unclear | Unclear | Unclear | 39 | 39 | *Jianpi Yifei* decoction + massage + conventional treatment | conventional treatment | Denervation potential |
|  | Unclear | Unclear | Unclear | Unclear |  |  |  |  | MUAPs amplitudes |
|  | Unclear | Unclear | Unclear | Unclear |  |  |  |  | Simple phase |

*MUNE, motor neuron number estimation; APB, abductor pollicis brevis; CMAP, compound muscle action potential; MUAP, motor unit action potential.*

*^a^ Reported in mean change and standard deviation for change from baseline.*

## Pharmacodynamic biomarker data

| **Study** | **Treatment Mean** | **Treatment SD** | **Control Mean** | **Control SD** | **Treatment N** | **Control N** | **Treatment** | **Control** | **Outcome measures** |
| --- | --- | --- | --- | --- | --- | --- | --- | --- | --- |
| Wang 2007 | 31.79 | 5.33 | 36.78 | 4.04 | 30 | 30 | *Jiweiling* injection | Riluzole | NSE |
|  | 0.5 | 0.28 | 0.79 | 0.46 |  |  |  |  | Asp |
|  | 0.46 | 0.27 | 0.95 | 0.65 |  |  |  |  | Glu |
|  | 29.41 | 13.24 | 34.11 | 9.81 |  |  |  |  | Gly |
|  | 32.53 | 15.13 | 32.18 | 6.96 |  |  |  |  | GABA |
| Wang 2009 | 2.5 | 1.08 | 3.07 | 2.5 | 100 | 25 | *Fuyuan Shengji* granule | Riluzole | IgA |
|  | 10.65 | 2.98 | 11.54 | 1.05 |  |  |  |  | IgG |
|  | 1.54 | 0.55 | 1.46 | 0.58 |  |  |  |  | IgM |
|  | 125.5 | 149.43 | 120.55 | 131.49 |  |  |  |  | CK |
|  | 16.97 | 15.02 | 12.69 | 3.77 |  |  |  |  | CK-MB |
|  | 138.63 | 41.73 | 124.54 | 24.42 |  |  |  |  | LDH |
| Chico 2018 | Unclear | Unclear | Unclear | Unclear | 15 | 21 | Curcumin | placebo | AOPPs |
|  | Unclear | Unclear | Unclear | Unclear |  |  |  |  | FRAP |
|  | Unclear | Unclear | Unclear | Unclear |  |  |  |  | T-SH |
|  | Unclear | Unclear | Unclear | Unclear |  |  |  |  | Lactate |

*NSE, neuron-specific enolase; Asp, aspartic acid; Glu, glutamic acid; Gly, glycine; GABA, gamma-amino butyric acid; Ig, immunoglobulin; CK, creatine kinase; CK-MB, creatine kinase isoenzyme; LDH, lactate dehydrogenase; AOPPs, advanced oxidation protein products; FRAP, ferric reducing antioxidant power; T-SH, total thiols.*

# Appendix 5. PRISMA 2020 checklist.

| **Section and Topic** | **Item #** | **Checklist item** | **Location where item is reported** |
| --- | --- | --- | --- |
| **TITLE** | | |  |
| Title | 1 | Identify the report as a systematic review. | Page 1, title |
| **ABSTRACT** | | |  |
| Abstract | 2 | See the PRISMA 2020 for Abstracts checklist. | Appendix 6 |
| **INTRODUCTION** | | |  |
| Rationale | 3 | Describe the rationale for the review in the context of existing knowledge. | Page 2, line 51 |
| Objectives | 4 | Provide an explicit statement of the objective(s) or question(s) the review addresses. | Page 3, line 79 |
| **METHODS** | | |  |
| Eligibility criteria | 5 | Specify the inclusion and exclusion criteria for the review and how studies were grouped for the syntheses. | Page 3, line 88 |
| Information sources | 6 | Specify all databases, registers, websites, organisations, reference lists and other sources searched or consulted to identify studies. Specify the date when each source was last searched or consulted. | Page 4, line 111 |
| Search strategy | 7 | Present the full search strategies for all databases, registers and websites, including any filters and limits used. | Page 4, line 130 |
| Selection process | 8 | Specify the methods used to decide whether a study met the inclusion criteria of the review, including how many reviewers screened each record and each report retrieved, whether they worked independently, and if applicable, details of automation tools used in the process. | Page 4, line 134 |
| Data collection process | 9 | Specify the methods used to collect data from reports, including how many reviewers collected data from each report, whether they worked independently, any processes for obtaining or confirming data from study investigators, and if applicable, details of automation tools used in the process. | Page 4, line 140 |
| Data items | 10a | List and define all outcomes for which data were sought. Specify whether all results that were compatible with each outcome domain in each study were sought (e.g. for all measures, time points, analyses), and if not, the methods used to decide which results to collect. | Page 3, line 105 |
|  | 10b | List and define all other variables for which data were sought (e.g. participant and intervention characteristics, funding sources). Describe any assumptions made about any missing or unclear information. | Page 4, line 140 |
| Study risk of bias assessment | 11 | Specify the methods used to assess risk of bias in the included studies, including details of the tool(s) used, how many reviewers assessed each study and whether they worked independently, and if applicable, details of automation tools used in the process. | Page 5, line 152 |
| Effect measures | 12 | Specify for each outcome the effect measure(s) (e.g. risk ratio, mean difference) used in the synthesis or presentation of results. | Page 5, line 172 |
| Synthesis methods | 13a | Describe the processes used to decide which studies were eligible for each synthesis (e.g. tabulating the study intervention characteristics and comparing against the planned groups for each synthesis (item #5)). | Page 5, line 170 |
|  | 13b | Describe any methods required to prepare the data for presentation or synthesis, such as handling of missing summary statistics, or data conversions. | Page 5, line 175 |
|  | 13c | Describe any methods used to tabulate or visually display results of individual studies and syntheses. | Page 5, line 183 |
|  | 13d | Describe any methods used to synthesize results and provide a rationale for the choice(s). If meta-analysis was performed, describe the model(s), method(s) to identify the presence and extent of statistical heterogeneity, and software package(s) used. | Page 5, line 169, 177 |
|  | 13e | Describe any methods used to explore possible causes of heterogeneity among study results (e.g. subgroup analysis, meta-regression). | Page 5, line 179 |
|  | 13f | Describe any sensitivity analyses conducted to assess robustness of the synthesized results. | Page 5, line 181 |
| Reporting bias assessment | 14 | Describe any methods used to assess risk of bias due to missing results in a synthesis (arising from reporting biases). | Page 5, line 161 |
| Certainty assessment | 15 | Describe any methods used to assess certainty (or confidence) in the body of evidence for an outcome. | Page 5, line 164 |
| **RESULTS** | | |  |
| Study selection | 16a | Describe the results of the search and selection process, from the number of records identified in the search to the number of studies included in the review, ideally using a flow diagram. | Page 6, line 194 |
|  | 16b | Cite studies that might appear to meet the inclusion criteria, but which were excluded, and explain why they were excluded. | Appendix 2 |
| Study characteristics | 17 | Cite each included study and present its characteristics. | Page 6, line 196 |
| Risk of bias in studies | 18 | Present assessments of risk of bias for each included study. | Page 7, line 235 |
| Results of individual studies | 19 | For all outcomes, present, for each study: (a) summary statistics for each group (where appropriate) and (b) an effect estimate and its precision (e.g. confidence/credible interval), ideally using structured tables or plots. | Appendix 4 |
| Results of syntheses | 20a | For each synthesis, briefly summarise the characteristics and risk of bias among contributing studies. | Page 20, Line 708; Page 24, line 720 |
|  | 20b | Present results of all statistical syntheses conducted. If meta-analysis was done, present for each the summary estimate and its precision (e.g. confidence/credible interval) and measures of statistical heterogeneity. If comparing groups, describe the direction of the effect. | Page 8, Line 260, 285; Page 10, line 333 |
|  | 20c | Present results of all investigations of possible causes of heterogeneity among study results. | Page 9, line 309 |
|  | 20d | Present results of all sensitivity analyses conducted to assess the robustness of the synthesized results. | Page 10, line 333 |
| Reporting biases | 21 | Present assessments of risk of bias due to missing results (arising from reporting biases) for each synthesis assessed. | Not applicable |
| Certainty of evidence | 22 | Present assessments of certainty (or confidence) in the body of evidence for each outcome assessed. | Page 10, line 369 |
| **DISCUSSION** | | |  |
| Discussion | 23a | Provide a general interpretation of the results in the context of other evidence. | Page 11, line 377 |
|  | 23b | Discuss any limitations of the evidence included in the review. | Page 11, line 393 |
|  | 23c | Discuss any limitations of the review processes used. | Page 13, line 485 |
|  | 23d | Discuss implications of the results for practice, policy, and future research. | Page 14, line 497 |
| **OTHER INFORMATION** | | |  |
| Registration and protocol | 24a | Provide registration information for the review, including register name and registration number, or state that the review was not registered. | Page 3, line 84 |
|  | 24b | Indicate where the review protocol can be accessed, or state that a protocol was not prepared. | No protocol |
|  | 24c | Describe and explain any amendments to information provided at registration or in the protocol. | Page 14, line 500 |
| Support | 25 | Describe sources of financial or non-financial support for the review, and the role of the funders or sponsors in the review. | Page 14, line 510 |
| Competing interests | 26 | Declare any competing interests of review authors. | When submitting |
| Availability of data, code and other materials | 27 | Report which of the following are publicly available and where they can be found: template data collection forms; data extracted from included studies; data used for all analyses; analytic code; any other materials used in the review. | When submitting |

# Appendix 6. PRISMA 2020 for abstracts checklist.

| **Section and Topic** | **Item #** | **Checklist item** | **Reported (Yes/No)** |
| --- | --- | --- | --- |
| **TITLE** | | |  |
| Title | 1 | Identify the report as a systematic review. | Yes |
| **BACKGROUND** | | |  |
| Objectives | 2 | Provide an explicit statement of the main objective(s) or question(s) the review addresses. | Yes |
| **METHODS** | | |  |
| Eligibility criteria | 3 | Specify the inclusion and exclusion criteria for the review. | Yes |
| Information sources | 4 | Specify the information sources (e.g. databases, registers) used to identify studies and the date when each was last searched. | Yes |
| Risk of bias | 5 | Specify the methods used to assess risk of bias in the included studies. | Yes |
| Synthesis of results | 6 | Specify the methods used to present and synthesise results. | Yes |
| **RESULTS** | | |  |
| Included studies | 7 | Give the total number of included studies and participants and summarise relevant characteristics of studies. | Yes |
| Synthesis of results | 8 | Present results for main outcomes, preferably indicating the number of included studies and participants for each. If meta-analysis was done, report the summary estimate and confidence/credible interval. If comparing groups, indicate the direction of the effect (i.e. which group is favoured). | Yes |
| **DISCUSSION** | | |  |
| Limitations of evidence | 9 | Provide a brief summary of the limitations of the evidence included in the review (e.g. study risk of bias, inconsistency and imprecision). | Yes |
| Interpretation | 10 | Provide a general interpretation of the results and important implications. | Yes |
| **OTHER** | | |  |
| Funding | 11 | Specify the primary source of funding for the review. | Yes |
| Registration | 12 | Provide the register name and registration number. | Yes |
